# Diverse Derivatives of Selenoureas: A Synthetic and Single Crystal Structural Study

**DOI:** 10.3390/molecules23092143

**Published:** 2018-08-25

**Authors:** Guoxiong Hua, David B. Cordes, Junyi Du, Alexandra M. Z. Slawin, J. Derek Woollins

**Affiliations:** School of Chemistry, University of St Andrews, St Andrews, Fife KY16 9ST, UK; gh15@st-andrews.ac.uk (G.H.); dbc21@st-andrews.ac.uk (D.B.C.); liangfengzhiyi@163.com (J.D.); Amzs@st-andrews.ac.uk (A.M.Z.S.)

**Keywords:** selenoureas, diselenazoles, selenoformamide, 1,3-selenazoles, Woollin’s Reagent

## Abstract

Reacting aroyl chlorides with an equivalent of potassium selenocyanate, followed by treating with an equivalent of 1,2,4-tri-tert-butylaniline at room temperature, resulted in the expected selenoureas and unusual diselenazoles. The selenation of selenourea by Woollins Reagent gave a new selenoformamide. Nucleophilic addition of selenoureas with acyl bromides led to the formation of new carbamimidoselenoates rather than the expected 1,3-selenazoles. The novel compounds prepared were characterised spectroscopically and crystallographically.

## 1. Introduction

Isoselenocyanates are useful precursors for the synthesis of selenium-containing heterocycles or heteroatom compounds [1,2,3]. Although the isolatable aryl isoselenocyanates have been characterised spectroscopically and crystallographically [1,4,5,6,7], the unstable aroyl isoselenocyanates can be only characterised via their further reaction with primary and secondary amines leading to the formation of *N*-benzoylselenoureas [8,9], for example, the aroyl isoselenocyanates generated in situ can be trapped with ethyl diazoacetate^9^ and diphenyldiazomethane [10]. The preparation of selenoureas is mainly carried out by reaction of isoselenocyanates with amines [11,12]. The selenoureas are generally considered to be the most efficient intermediates for the introduction of selenium into heterocycles and heteroatom compounds, as they are conveniently prepared and relatively stable [4,7,13,14,15,16,17,18,19,20]. As a part of our ongoing projects focused on the synthesis and characterisation of selenium-containing heteroatom systems herein we report the formation of new selenoureas and diselenazoles via the reactions of aroyl chlorides, potassium selenocyanate, and the bulky amine 1,2,4-tri-tert-butylaniline, the further selenation by Woollins Reagent (WR) and nucleophilic addition with acyl bromides, and five single crystal X-ray structures.

## 2. Results and Discussion

### 2.1. Synthesis and Characterization

**Scheme 1:**
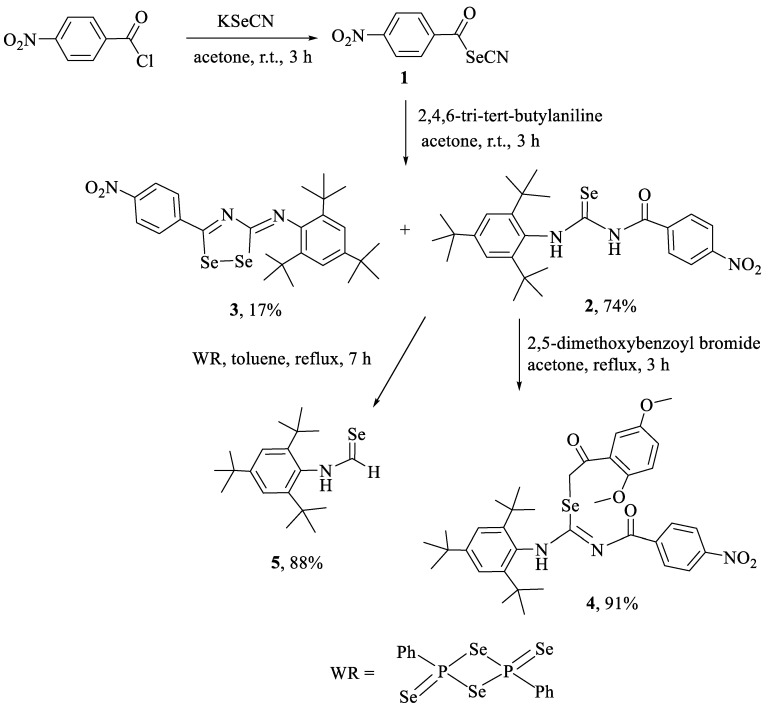
Synthesis of compounds **1**–**5**.

As shown in Scheme 1, the reaction of 4-nitrobenzoyl chloride with KSeCN led to an intermediate **1**, the latter was treated in situ with 1,2,4-tri-tert-butylaniline to give selenourea **2** as a major product in 74% yield and diselenazole **3** as an unexpected by-product in 17% yield. We found that the similar compound 3-benzoylimino-5-(morpholin-4-yl)-1,2,4-diselenazole was prepared unexpectedly in ca. 20% yield as an uncharacterized side-product by the one-pot reaction of benzoyl chloride, potassium selenocyanate, and ethyl diazoacetate after treatment with morpholine. As shown in Scheme 1, selenourea **2** reacted further with 2,5-dimethoxybenzoyl bromide in refluxing acetone resulting in the unexpected 2-(2,5-dimethoxyphenyl)-2-oxoethyl-*N′*-(4-nitrobenzoyl)-*N*-(2,4,6-tri-tert-butylphenyl)carbamimidoselenoate (**4**) in 91% isolated yield rather than five-membered heterocycle (2,5-dimethoxyphenyl)(4-[4-nitrophenyl]-2-[{2,4,6-tri-tert-butylphenyl}amino]-1,3-selenazol-5-yl)methanone. The results suggest that the steric effect controlled the formation of the final product in this reaction. 

Interestingly, the selenation of selenourea **2** by using WR led to the new selenoformamide **5** in 88% yield as a unique isolatable product, along with phosphorus-containing byproducts derived from WR. Although the sterically demanding selenoformamide **5** was never produced previously, its analogues have been prepared from the reaction of amides and Al-E [a mixture of (^i^Bu_2_AlE)_2_ and (^i^Bu_2_AlE)_n_, E = S, Se and Te] reagents [21,22] or direct thionation and selenation of amides using elemental sulfur and selenium and hydrochlorosilanes in the presence of amines [23]. 

**Scheme 2:**
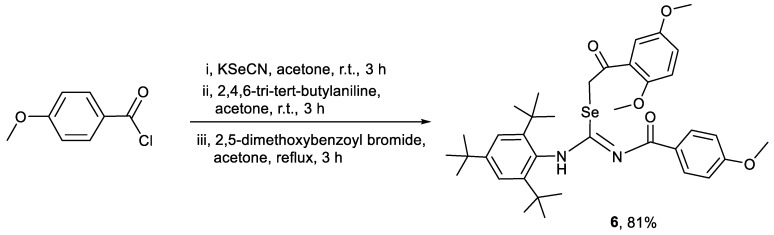
Synthesis of compound **6**.

**Scheme 3:**
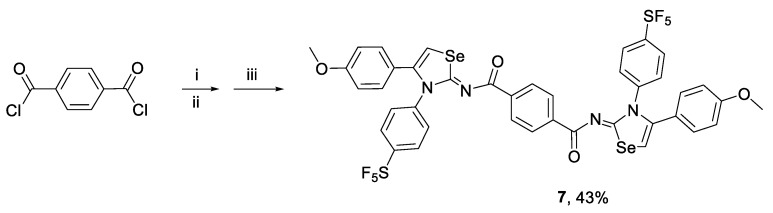
Synthesis of compound **7**. i, KSeCN, acetone, r.t., 3 h; ii, 4-pentafluorosulfanyl-aniline, acetone, r.t., 3 h; iii, 4-methoxybenzoyl bromide, acetone, reflux, 3 h.

Similarly, one-pot reaction of 4-methoxybenzoyl chloride with an equivalent amount of KSeCN and 2,4,6-tri-tert-butylaniline and 2-bromo-2′,5′-dimethoxyacetophenone under the same condition as above gave the expected 2-(2,5-dimethoxyphenyl)-2-oxoethyl-*N′*-(4-methoxybenzoyl)-*N*-(2,4,6-tri-tert-butylphenyl)-carbamimidoselenoate (**6**) in 81% isolated yield after work-up (Scheme 2). However, one-pot reaction of terephthaloyl dichloride with one equivalent of KSeCN and 4-pentafluorosulfanylaniline and 4-methoxybenzoyl bromide under the same reaction conditions afforded 1,4-*bis*[*N*-(4-(4-methoxyphenyl)-3-(4-(pentafluoro-l6-sulfanyl)phenyl)-1,3-selenazol-2(3H)-ylidene)]-terephthal-amide **7** in 43% yield rather than the analogue of non-cyclic carbamimidoselenoate **4** or **6** (Scheme 3). The results showed that the choice of the amine is the key for the formation of final product, where the bulky amine 2,4,6-tri-tert-butylaniline is preferable for the formation of non-cyclic carbamimidoselenoate whilst the non-bulky amine favors to the formation of 1,3-selenazole ring. 

The structures of new compounds **2**–**7** were assigned based on their spectroscopic data (see Supplementary Materials) and mass spectral analyses; the [M]^+^ or [M + H]^+^ peaks in their high-resolution mass spectra and isotopic distribution patterns being perfectly agreement with the calculated ones in all compounds. The chemical shifts of carbonyl carbon (C=O) in the ^13^C-NMR spectra (165.9 ppm for compound **4**, 167.4 and 164.1 ppm for compound **6**, and 165.9 and 165.4 ppm for compound **7**) are similar to the value for the amide carbonyl carbon in compound **2** (165.0 ppm), which is in the range found in the similar aroyl-acyl selenoureas 165.3 ± 2.14 ppm [21]. The signals in the ^77^Se NMR are shifted downfield from 452.6 ppm for selenamide **2** to 640.0 and 630.5 ppm for 1,2,4-diselenol-3-imine **3**, 610.5 ppm for selenoformamide **5**, 590.7 ppm for carbamimdoselenoate **6**, and 617.7 and 614.3 ppm for compound **7**; and shifted upfield to 396.9 ppm for carbamimdoselenoate **4**. The proton resonances are only observed to be *^t^*Bu groups attached to phenyl rings, phenyl ring, and nitrogen atoms in **2**. In compound **3**, two selenium signals were observed to be not equal with ca. 10 ppm difference between them (*δ*_Se_ 640.0 and 630.5 ppm, respectively); and there is no proton found to be attached to the nitrogen atoms, confirming the proposed formulation. In compounds **4** and **6**, there are proton signals for CH_2_ and OCH_3_ groups together with the resonances from the attached *^t^*But groups on phenyl rings, phenyl ring, and NH groups. In the case of compound **5**, the chemical shift (*δ*_H_ 10.28 ppm) of the selenoamido hydrogen atom in the ^1^H-NMR spectrum is much bigger than the value of the selenoamido proton in compound **2** (*δ*_H_ 9.67 ppm). It is worth noting that a doublet signal for the selenoamido hydrogen atom is observed, however, only singlet signals for the selenoamido hydrogen atom is found in compound **5**, revealing the big influence of the intramolecular and intermolecular hydrogen bonding. 

### 2.2. Single Crystal Structure Analysis

**Figure 1:**
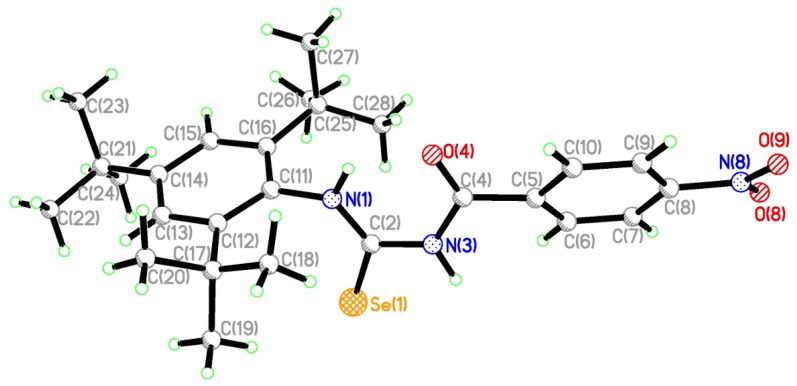
Single crystal X-ray structure of compound **2** (hydrogens represented as small green spheres). C(11)-N(1) 1.438(2), N(1)-C(2) 1.318(2), Se(1)-C(2), 1.830(2), C(2)-N(3) 1.388(2), N(3)-C(4) 1.379(2), C(4)-O(4) 1.217(2), C(4)-C(5) 1.491(2); C(11)-N(1)-C(2) 123.90(13), N(1)-C(2)-Se(1) 125.61(12), Se(1)-C(2)-N(3) 116.70(11), C(2)-N(3)-C(4) 127.43(14), N(3)-C(4)-O(4) 122.77(15), O(4)-C(4)-C(5) 121.74(14).

**Figure 2:**
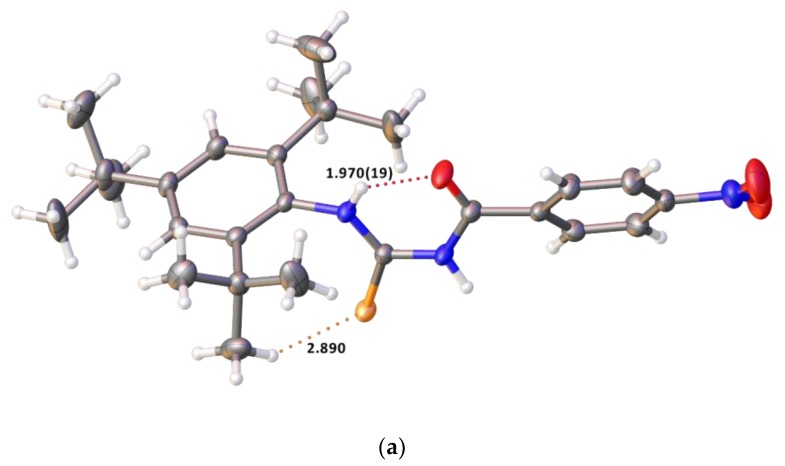

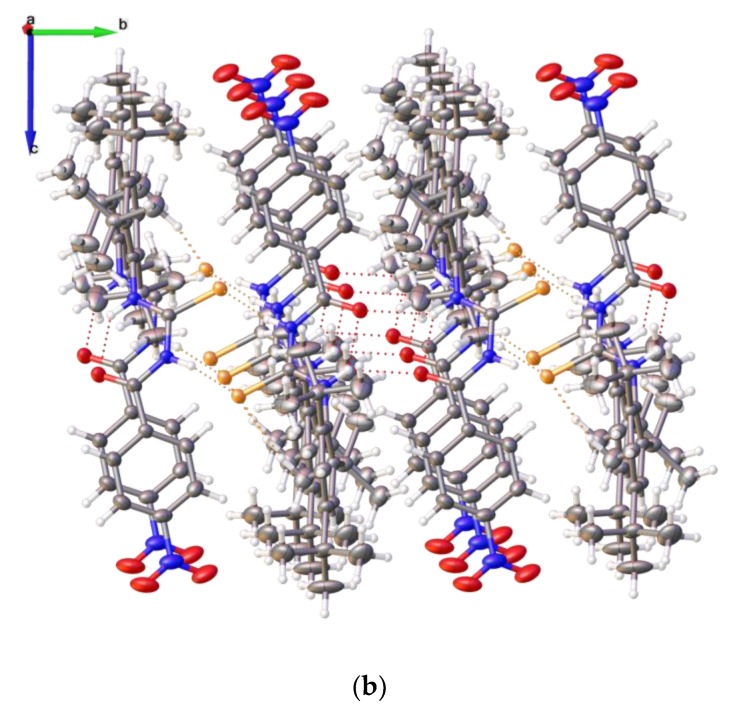
(**a**): intramolecular interactions seen in **2**; (**b**): supramolecular structure in **2**. Atoms colors are the same as in Figure 1 except hydrogen, which is white.

**Figure 3:**
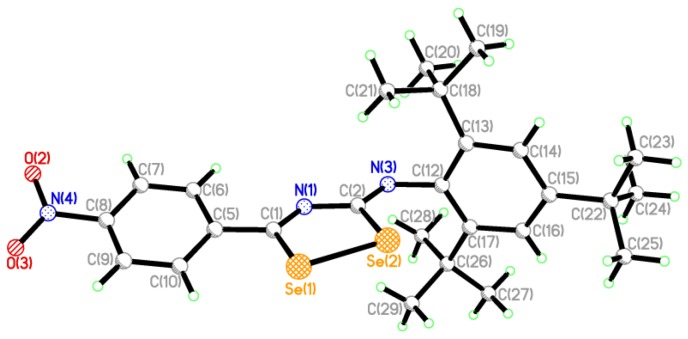
Single crystal X-ray structure of compound **3** (hydrogens represented as small green spheres). Se(1)-Se(2) 2.3118(12)[2.3116(12)], Se(1)-C(1) 1.925(8)[1.910(7)], Se(2)-C(2) 1.970(8)[1.952(7)], C(1)-N(1) 1.272(9)[1.267(10)], C(2)-N(1) 1.383(10)[1.398(10)], C(2)-N(3) 1.273(10)[1.270(10)], N(3)-C(12) 1.425(10)[1.436(9)]; C(1)-Se(1)-Se(2) 89.6(2)[89.3(3)], C(2)-Se(2)-Se(1) 90.1(3)[90.1(2)], Se(1)-C(1)-N(1) 122.6(6)[124.0(6)], Se(2)-C(2)-N(1) 117.1(6)[117.7(6)], C(1)-N(1)-C(2) 120.5(7)[118.7(7)], Se(2)-C(2)-N(3) 120.2(6)[122.2(6)], N(1)-C(2)-N(3) 122.6(7)[120.1(7)].

**Figure 4:**
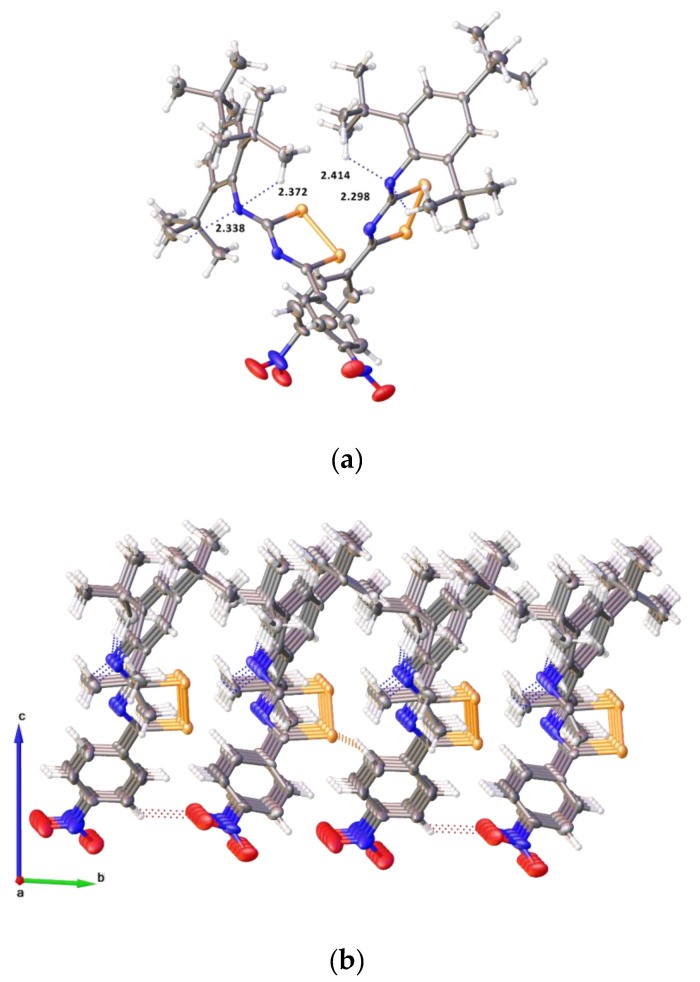
(**a**): intramolecular interactions seen in **3**; (**b**): supramolecular structure in **3**. Atoms colors are the same as in Figure 3 except hydrogen, which is white.

**Figure 5:**
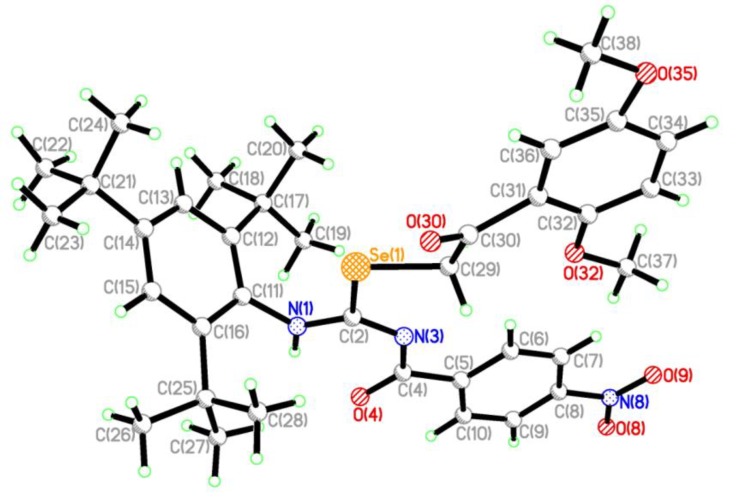
Single crystal X-ray structure of compound **4**. N(1)-C(2) 1.326(5)[1.319(5), 1.328(5)], C(2)-Se(1) 1.895(4)[1.898(4), 1.900(4)], C(2)-N(3) 1.331(5)[1.329(5), 1.323(5)], Se(1)-C(29) 2.021(9)[1.943(4), 1.955(4)], C(29)-C(30) 1.483(13)[1.497(7), 1.489(6)], C(30)-O(30) 1.240(12)[1.187(6), 1.215(5)], N(3)-C(4) 1.350(5)[1.356(5), 1.353(5)], C(4)-O(4) 1.232(5)[1.232(5), 1.241(5)]; N(1)-C(2)-Se(1) 115.8(3)[115.7(3), 115.6(3)], C(2)-Se(1)-C(29) 93.9(3)[96.22(18), 97.52(16)], Se(1)-C(29)-C(30) 108.3(6)[107.4(3), 107.4(3)], C(29)-C(30)-O(30) 116.4(9)[119.4(5), 119.6(3)], Se(1)-C(2)-N(3) 117.9(3)[117.6(3), 118.3(3)], N(1)-C(2)-N(3) 126.3(4)[126.7(4), 126.1(4)], C(2)-N(3)-C(4) 120.6(3)[119.5(3), 119.7(3)], N(3)-C(4)-O(4) 126.6(4)[127.3(3), 127.9(4)].

**Figure 6:**
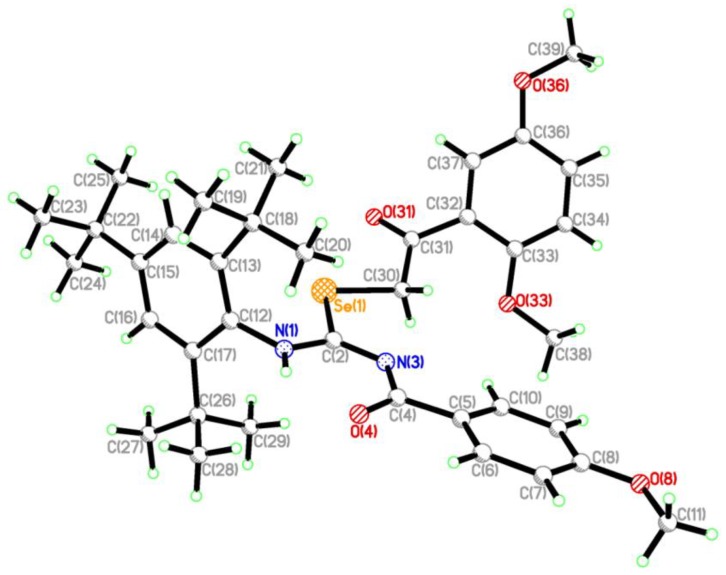
Single crystal X-ray structure of compound **6**. N(1)-C(2) 1.327(3), C(2)-Se(1) 1.918(3), Se(1)-C(30) 1.945(3), C(30)-C(31) 1.496(4), C(31)-O(31) 1.220(3), C(2)-N(3) 1.319(3), N(3)-C(4) 1.362(3), C(4)-O(4) 1.239(3); N(1)-C(2)-Se(1) 114.49(18), N(1)-C(2)-N(3) 127.3(2), C(2)-Se(1)-C(30) 95.82(11), Se(1)-C(30)-C(31) 108.14(18), C(30)-C(31)-O(31) 120.6(3), Se(1)-C(2)-N(3) 118.25(19), C(2)-N(3)-C(4) 120.3(2), N(3)-C(4)-O(4) 125.9(2).

**Figure 7:**
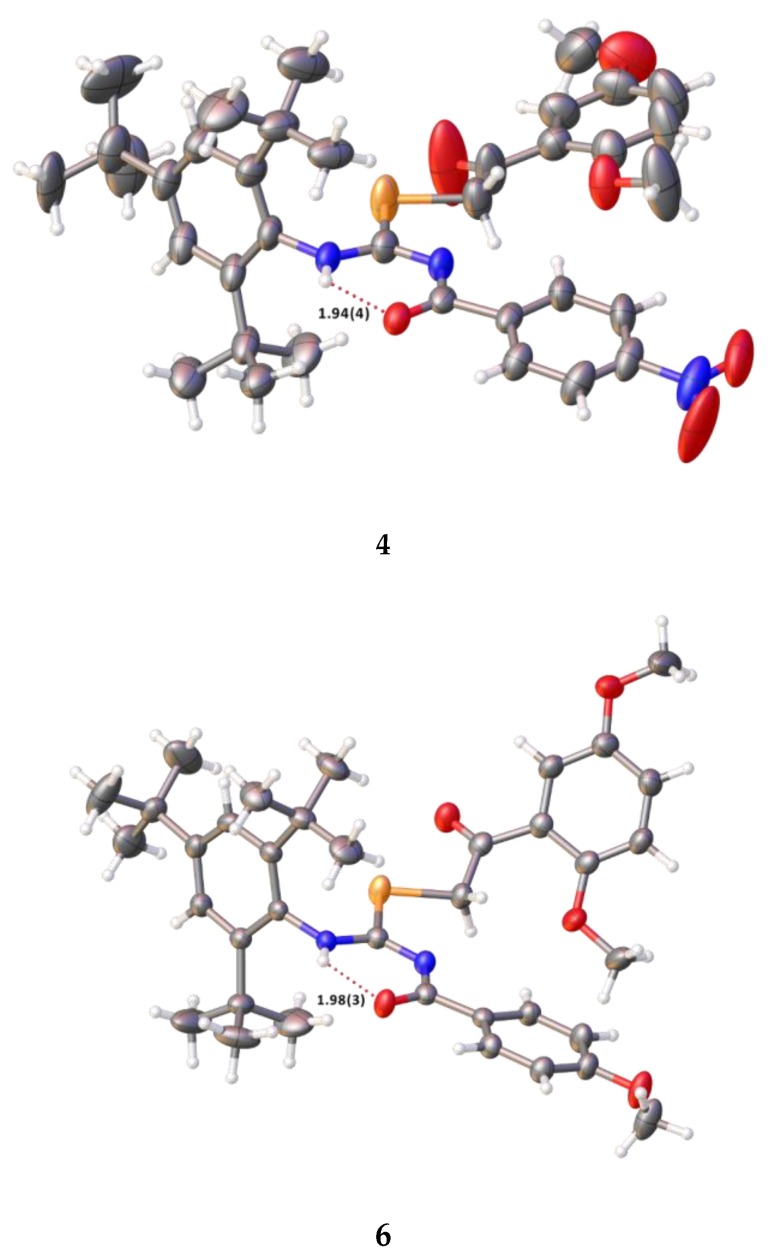
Strong intramolecular N–H∙∙∙O hydrogen bonding in compounds **4** (only one independent molecule shown) and **6**. Atoms colors are the same as in Figure 5 and Figure 6 except hydrogen, which is white.

**Figure 8:**
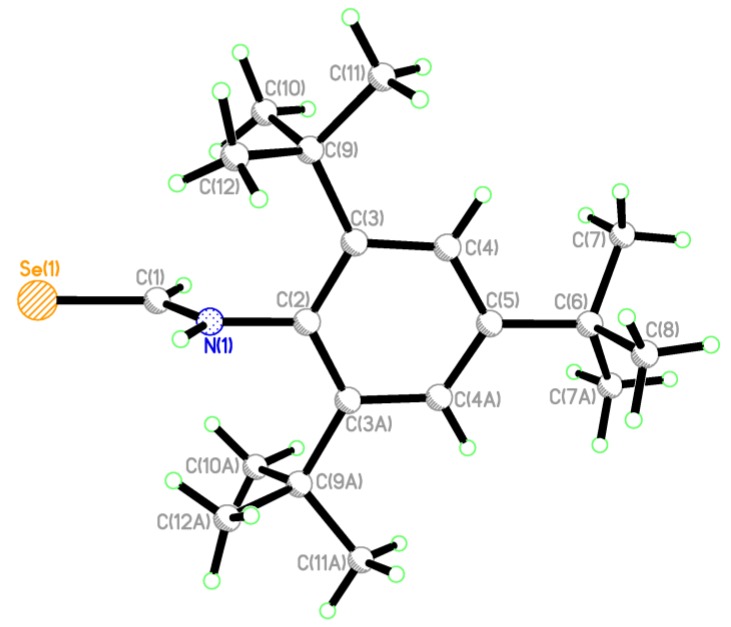
X-ray single crystal structure of **5**. Se(1)-C(1) 1.807(5), N(1)-C(1) 1.324(7), N(1)-C(2) 1.448(6); Se(1)-C(1)-N(1) 125.1(4), C(1)-N(1)-C(2) 125.1(4); Se(1)-C(1)-N(1)-C(2) 180.0.

Crystals of the compounds **2**–**6** suitable for X-ray crystallographic analysis were grown by slow evaporation of the chloroform or dichloromethane solution of the compound in air at room temperature. The crystal structures of compounds **2**–**6** are shown in Figure 1, Figure 2, Figure 3, Figure 4, Figure 5, Figure 6, Figure 7 and Figure 8. The crystallographic data and structure refinement details are depicted in Table 1. There is one molecule of the compound and one molecule of chloroform in the asymmetric unit in **2**, two molecules of the compound and highly disordered dichloromethane (removed using PLATON SQUEEZE [24]) in **3**, and three symmetry-independent molecules of the compound in the case of **4** in the asymmetric unit. However, there is a single molecule of the compound in the asymmetric unit in **5** and **6**. In the structure of **2** as shown in Figure 1, the O(4)-C(4)-N(3)-C(2)-Se(1)-N(1) is near planar with the maximum deviation being 0.072 Å for atom N(3); the orientations of the two adjacent phenyl rings are quite different from the mean plane of O(4)-C(4)-N(3)-C(2)-Se(1)-N(1) with dihedral angles of 31.2 and 82.4°, respectively. The benzylic carbon C(4) atom is located nearly in the same plane as the N(1)-C(2)-N(3) plane. As a result, we suggest that the lone pair electrons on N(1) and N(3) atoms are almost perpendicular to the C=Se group. The similar C–N single bond lengths [N(1)-C(2) 1.318(2) Å, C(2)-N(3) 1.388(2) Å, N(3)-C(4) 1.379(2) Å] in **2** are significantly shorter than the typical single bond length [1.47Å] and the C=O and C=Se double bond distances [C(4)-O(4) 1.217(2) Å, Se(1)-C(2), 1.830(2) Å] indicate a delocalized π-system [24]. 

In addition, each molecule is connected to another molecule through intermolecular N–H···O and weaker N–H···Se hydrogen bonding, accompanied by the strong intramolecular N–H···O hydrogen bonding and weak intramolecular C–H···Se hydrogen bonding, results in a dimeric arrangement with two molecules in *trans* conformation **2**. Both intramolecular and intermolecular N–H···O hydrogen bonds are not linear with the similar N–H···O angles of 135.1(18)° and 140.6(17)°, respectively, however, the intramolecular H···O distance [1.971(18) Å] is significantly shorter than the intermolecular H···O distance [2.370(18) Å], suggesting that the formation of the intramolecular N–H···O hydrogen bond is strongly favored over the corresponding intermolecular one. Both the intramolecular and intermolecular C–H···Se hydrogen bonding [H···Se distance of 2.890 Å, C–H···Se angle of 151.05°, and H···Se distance of 2.662(16) Å, N–H···Se angle of 157.3(17)°] are rather weaker, compared to the N–H···O hydrogen bonding. A combination of strong intramolecular and intermolecular N–H···O hydrogen bonding, complemented by weaker interactions involving Se results in the supramolecular structure as shown in Figure 2, in which the amide N–H, the best H-donor, is involved in both intramolecular and intermolecular hydrogen bonding. 

As shown in Figure 3, in the structure of **3**, the newly formed diselenazole rings are nearly planar; the maximum deviation for the azole rings thorough all ring atoms being 0.009 Å and 0.021 Å for the two independent molecules. Both molecules are quite similar to each other in all distances and angles; the two aryl rings are not coplanar with the mean plane of the diselenazole ring due to the steric effect, with the dihedral angles between the two aryl rings and the mean plane of the diselenazole ring of 23.29 [9.03] and 89.72 [89.98]°, respectively (Figure 4a). The four C–N bond lengths are different [two endocyclic C(1)-N(1) 1.272(9)[1.267(10)] Å and C(2)-N(1) 1.383(10)[1.398(10)] Å and two exocyclic C(2)-N(3) 1.273(10)[1.270(10)] Å and C(12)-N(3) 1.425(10)[1.436(9)] Å], which are shorter than the typical C–N single bond length [1.47 Å] indicating a limited delocalized *π*-system which is highly influenced by steric conformation. The Se–Se bond length {2.3118(12)[2.3116(12)] Å} is somewhat shorter than the sum of the covalent radii (2.34 Å), and significantly shorter than that in 2-benzoylamino-5-diethylamino-1,6,6aλ^4^-triselena-3,4-diazapentalene [2.514(1)-2.641 (1) Å] [25], but clearly shorter than the sum of the van der Waals radii (3.8 Å). In supramolecular structure of **3**, the weak intramolecular C–H∙∙∙N hydrogen bonds were observed with H∙∙∙N distances ranging from 2.298 to 2.414 Å with the corresponding angles 121.58 to 126.04°. The intermolecular C–H∙∙∙O and C–H∙∙∙Se interactions led to the formation of supramolecular framework (Figure 4).

Not surprisingly, the structures of **4** and **6** have a similar conformation (Figure 5 and Figure 6). The Se(1)-N(1)-C(2)-N(3)-C(4)-O(4) atoms are nearly coplanar with the maximum deviations of −0.147 [0.028 and 0.026] Å in **4** and 0.027 Å in **6**, along with the O(30) atom being 1.142 [0.632 and 0.406] Å in **4** and 0.843 Å in **6** above the mean plane. The dihedral angles between the Se(1)-N(1)-C(2)-N(3)-C(4)-O(4) mean plane and two phenyl rings are 21.28 [10.61 and 17.63] and 80.69 [83.46 and 89.27]° in **4** and 7.47 and 86.25° in **6**. Three consecutive C-N bond lengths {N(1)-C(2) 1.326(5) [1.319(5), 1.328(5)], C(2)-N(3) 1.331(5) [1.329(5), 1.323(5)], and N(3)-C(4) 1.350(5)[1.356(5), 1.353(5)] Å in **4** and N(1)-C(2) 1.327(3), C(2)-N(3) 1.319(3), and N(3)-C(4) 1.362(3) in **6**} are similar to each other, however, these values are significantly shorter than the usual single bond length of 1.47 Å [26], indicating the delocalization of π-electrons and the lone pair electrons on N1 and N3 atoms and it is a rare sample of antiperiplanar carbamimidoselenoate. The single C(2)-Se(1) bond lengths {1.895(4) [1.898(4), 1.900(4)] Å in **4** and 1.918(3) Å in **6**} is significantly shorter than the typical single Se(1)-C(29) bond distances {2.021(9) [1.943(4), 1.955(4)] Å in **4** and 1.945(3) Å in **6**}, however, both bond lengths are marginally shorter than in comparison with the range of values found for the diselenides (1.91–1.97 Å) [27], revealing the partial double-bond character in the delocalized π-system of the adjoining C=N. 

Further stabilization of the crystal structures in compounds **4** and **6** is achieved by strong intramolecular N–H∙∙∙O hydrogen bond between the NH group and O atoms of amide groups with bond lengths O···H of 1.88(4), 1.94(4), 2.01(4) Å in **4** and 1.98(3) Å in **6**, and angles at H of 134(4), 126.(4), 123.(3) ]° in **4**, and 126(2)° in **6** (Figure 7). Furthermore, two molecules of three independent molecules link via weak intermolecular C–H∙∙∙O hydrogen bonding in **4**.

**Figure 9:**
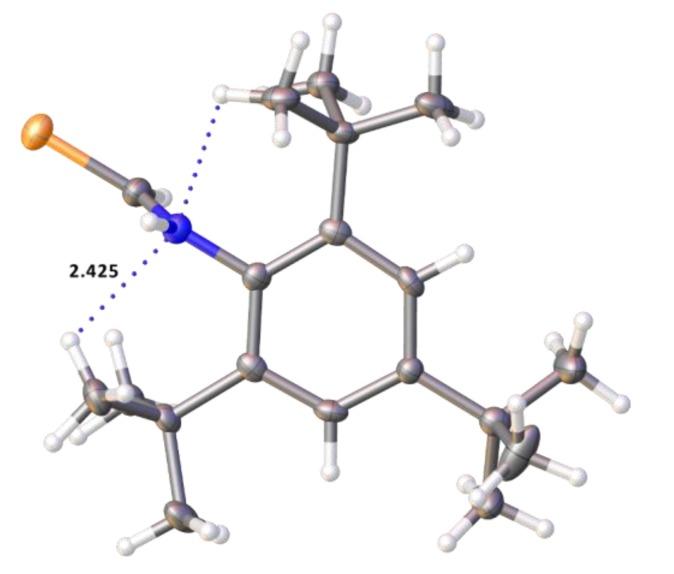

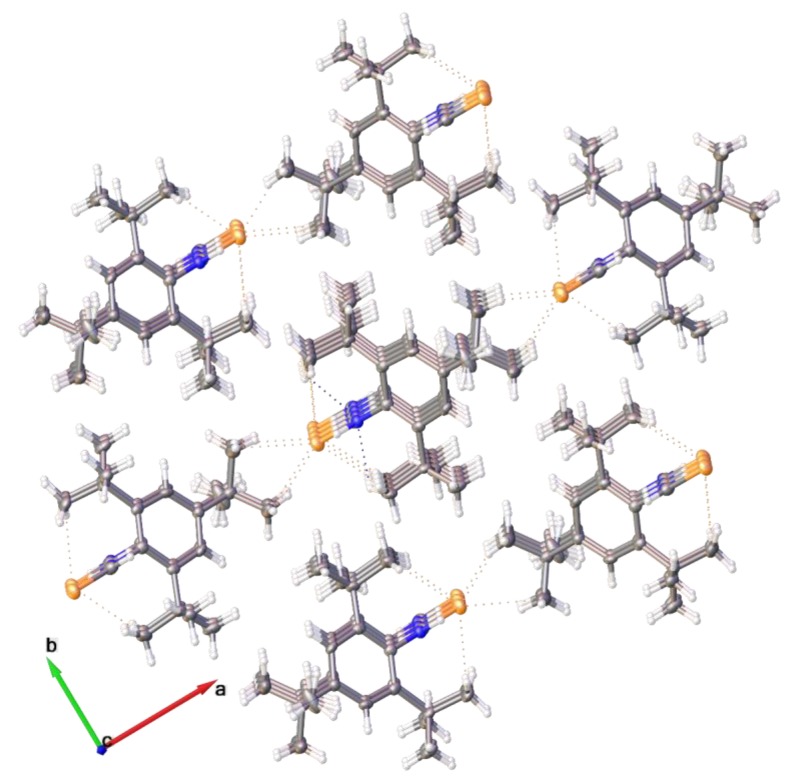
(**a**): intramolecular interactions seen in **5**; (**b**): supramolecular structure in **5**. Atoms colors are the same as in Figure 8 except hydrogen, which is white.

The single crystal structure of **5** (Figure 8) adopts a highly symmetrical conformation: the C8, C6, C5, C2 atoms are perfectly co-planar with the methaneselenoamide moiety (SeCN unit); the SeCN unit is perpendicular to the phenyl ring, and three substituted carbons attaching to the phenyl ring are also co-planar with the phenyl ring. The structure is different to the analogue structure of *N*-(2,4,6-trimethylphenyl)formamide [68.06(10)°] [28], since there is clear steric effect of three substituted *^t^*Bu groups on the phenyl ring. The double C=Se bond length [1.807(5) Å] is significantly shorter than that in selenoamides [1.815(5) to 1.856(4) Å], whilst the C–N bond length [1.324(7) Å] falling in the range of 1.270(7) to 1.324(8) Å for C–N bond in selenoamides [29,30]. It is interesting to note that there is weak intramolecular C–H∙∙∙N hydrogen bonding which may influence the conformation in Figure 9. It is also clear that the weak intermolecular C–H∙∙∙Se hydrogen bonding and π–π stacking are important features of the solid-state packing of the supramolecular structure.

## 3. Materials and Methods

### 3.1. General

Unless otherwise stated, all reactions were carried out under on oxygen free nitrogen atmosphere using pre-dried solvents and standard Schlenk techniques, subsequent chromatographic and work up procedures were performed in air. ^1^H (400.1 MHz), ^13^C (100.6 MHz), and ^31^P-{^1^H} (162.0 MHz) NMR spectra were recorded at 25 °C (unless stated otherwise) on Bruker Advance II 400s (Bruker, Blue Lion Biotech, Carnation, WA, USA). IR spectra were recorded as KBr pellets in the range of 4000–250 cm^−1^ on a Perkin-Elmer 2000 FTIR/Raman spectrometer (Perkin-Elmer, Beaconfield, UK). Mass spectrometry was performed by the EPSRC National Mass Spectrometry Service Centre, Swansea. X-ray diffraction data for compounds **2**, **4**, and **6** were collected at −100(1) °C using a Rigaku FR-X Ultrahigh-Brilliance Microfocus RA generator (Mo Kα radiation, confocal optic) with XtaLAB P200 diffractometer (The Woodlands, TX, USA). Data for compounds **3** and **5** were collected at −148(1) °C using the St Andrews Automated Robotic Diffractometer (STANDARD, The Woodlands, TX, USA) [31], a Rigaku sealed-tube generator (Mo Kα radiation, SHINE monochromator,) and Saturn 724 CCD system, coupled with a Microglide goniometer head and an ACTOR-SM robotic sample changer. For all compounds, at least a full hemisphere of data was collected using ω scans. Data for all compounds analysed were collected and processed (including correction for Lorentz, polarization, and absorption) using CrystalClear (Rigaku) [32,33]. Structures were solved by direct (SIR92 [34], SIR2004 [35] or SIR2011 [36]) or Patterson (PATTY [37]) methods and expanded using Fourier techniques. Non-hydrogen atoms were refined anisotropically. Hydrogen atoms were refined using the riding model, except for NH hydrogens which were located from the difference Fourier map and refined isotropically subject to a distance restraint, and with riding thermal parameters. Disorder in *^t^*Bu groups was apparent in **2** and **4**, as well as disorder in the CHCl_3_ solvent in **2**, in one of the NO_2_ groups in **3**, and in one (dimethoxy)benzoylmethyl group in **4**. In all cases this was treated by splitting the disordered atoms, and refining them (anisotropically for the major component only, except for Cl) with partial atomic occupancies summing to one across both components of each disordered group. Restraints to bond distances and angles were required in a number of cases, particularly in the minor component of a disordered group. Individual disordered atoms were constrained to have similar thermal parameters across both disordered components, and in some cases further restraints to thermal parameters were required. In **3**, early attempts at modelling had revealed highly disordered partial solvent molecules, likely CH_2_Cl_2_. No chemically sensible disorder model could be obtained for these, so the PLATON SQUEEZE [38] routine was used to remove the contribution of the poorly ordered electron density. All calculations were performed using the CrystalStructure [39] crystallographic software package except for refinement, which was performed using SHELXL2018 [40]. Some figures were created using Olex2 [41]. CCDC 1855800-1855804 contain the supplementary crystallographic data for this paper. The data can be obtained free of charge from The Cambridge Crystallographic Data Centre via www.ccdc.cam.ac.uk/structures.

### 3.2. Synthesis

#### 3.2.1. Synthesis of Compounds **2** and **3**

To a solution of 4-nitrobenzoyl chloride (0.370 g, 2.0 mmol) in dry acetone (30 cm^3^) was added dropwise a solution of KSeCN (0.288 g, 2.0 mmol) in dry acetone (10 mL) at room temperature. 2,4,6-tri-tert-Butylaniline (0.523 g, 2.0 mmol) was added. After the mixture was stirred at room temperature for 3 h, then the suspension was stirred in the darkness at room temperature for another 18 h. After removing solvent in vacuo, the residue was purified by silica gel column (eluted by dichloromethane) to give the title products **2** and **3**.

*4-Nitro-N-((2,4,6-tri-tert-butylphenyl)carbamoselenoyl)benzamide* (**2**). Yellow solid (0.765 g, 74%). M.p. 120–122 °C. Selected IR (KBr, cm^−1^): 1675, 1601, 1521, 1363, 1342, 1258, 1153, 1110, 1012, 862, 834, 755, 710, 653, 582. ^1^H-NMR (CDCl_3_, δ), 9.67 (s, 1H), 8.38 (d, *J*(H,H) = 9.1 Hz, 2H), 8.14 (d, *J*(H,H) = 9.1 Hz, 2H), 7.42 (s, 2H), 5.28 (s, 1H), 1.44 (s, 18H), 1.34 (s, 9H) ppm. ^13^C-NMR (CDCl_3_, δ), 183.7, 165.0, 150.9, 150.3, 145.9, 136.8, 132.7, 129.2, 124.4, 124.3, 37.1, 35.1, 32.7, 31.5 ppm. ^77^Se-NMR (CDCl_3_, δ), 452.6 ppm. HRMS (EI^+^, *m/z*): found 517.1840 [M]^+^, calculated mass for C_26_H_35_N_3_O_3_Se: 517.1844. Elemental analysis: Found C, 60.36; H, 6.77; N, 8.27; C_26_H_35_N_3_O_3_Se requires C, 60.46; H, 6.83; N, 8.14.

*5-(4-Nitrophenyl)-N-(2,4,6-tri-tert-butylphenyl)-3H-1,2,4-diselenazol-3-imine* (**3**). Orange solid (0.200 g, 17%). M.p. 100–102 °C. Selected IR (KBr, cm^−1^): 2278, 1607, 1530, 1476, 1435, 1394, 1361, 1346, 1237, 1072, 879, 847, 614. ^1^H-NMR (CDCl_3_, δ), 8.23 (d, *J*(H,H) = 8.2 Hz, 2H), 8.14 (d, *J*(H,H) = 8.2 Hz, 2H), 7.34 (s, 1H), 7.10 (s, 1H), 1.36 (s, 9H), 1.31 (s, 1H), 1.29 (s, 9H), 1.28 (s, 4H), 1.19 (s, 4H) ppm. ^13^C-NMR (CDCl_3_, δ), 179.9, 174.7, 151.0, 149.0, 141.9, 139.4, 134.3, 130.4, 125.0, 122.4, 37.2, 35.5, 35.4, 32.5, 32.2, 32.1, 30.8 ppm. ^77^Se-NMR (CDCl_3_, δ), 640.0 and 630.5 ppm. HRMS (APCI^+^, *m/z*): found 580.0978 [M + H]^+^, calculated mass for C_26_H_33_N_3_O_2_Se_2_H: 580.0981. Elemental analysis: Found C, 54.16; H, 5.77; N, 7.20; C_26_H_33_N_3_O_2_Se_2_ requires C, 54.08; H, 5.76; N, 7.28.

#### 3.2.2. Synthesis of 2-(2,5-dimethoxyphenyl)-2-oxoethyl-*N′*-(4-nitrobenzoyl)-*N*-(2,4,6-tri-tert-butylphenyl)-carbamimidoselenoate (**4**)

To a solution of 4-nitro-*N*-((2,4,6-tri-tert-butylphenyl)carbamoselenoyl)benzamide (0.517 g, 1.0 mmol) in dry acetone (10 cm^3^) was added 2-bromo-2′,5′-dimethoxyacetophenone (0.258 g, 1.0 mmol). The mixture was stirred at room temperature for 24 h, then refluxed for 3 h. Upon cooling to room temperature and removing the solvent, the residue was shaken with water and extracted with dichloromethane (30 cm^3^ × 3), the combined organic layers were dried over MgSO_4_. After removing solvent, the final residue was purified by silica gel column (eluted by 1:5 ethyl acetate–hexane) to give the title product **4** as dark yellow solid (0.630 g, 91%). M.p. 145–147 °C. Selected IR (KBr, cm^−1^): 1729, 1580, 1527, 1497, 1338, 1280, 1217, 1154, 1106, 1047, 1014, 877, 806, 719. ^1^H-NMR (CD_2_Cl_2_, δ), 8.34 (d, *J*(H,H) = 8.8 Hz, 2H), 8.18 (d, *J*(H,H) = 8.8 Hz, 2H), 8.11 (s, 1H), 7.57 (s, 1H), 7.37 (s, 2H), 7.29 (d, *J*(H,H) = 8.5 Hz, 1H), 7.05 (d, *J*(H,H) = 8.5 Hz, 1H), 3.86 (s, 3H), 3.89 (s, 3H), 2.59 (s, 2H), 1.33–1.26 (m, 27H) ppm. ^13^C-NMR (CD_2_Cl_2_, δ), 179.6, 174.7, 165.9, 154.4, 153.7, 152.0, 151.3, 148.8, 131.7, 131.4, 125.1, 124.3, 123.7, 122.6, 121.2, 115.5, 57.1, 56.5, 40.2, 37.7, 35.8, 33.4, 33.2, 32.9, 31.9 ppm. ^77^Se-NMR (CD_2_Cl_2_, δ), 396.9 ppm. HRMS (EI^+^, *m/z*): found 694.2389 [M − H]^+^, calculated mass for C_36_H_44_N_3_O_6_Se: 694.2393. Elemental analysis: Found C, 63.67; H, 6.31; N, 6.23; C_36_H_44_N_3_O_6_Se requires C, 62.24; H, 6.53; N, 6.05.

#### 3.2.3. Synthesis of 2-(2,5-dimethoxyphenyl)-2-oxoethyl-*N′*-(4-methoxybenzoyl)-*N*-(2,4,6-tri-tert-butylphenyl)-carbamimidoselenoate (**6**)

To a solution of 4-methoxybenzoyl chloride (0.340 g, 2.0 mmol) in dry acetone (50 cm^3^) was added dropwise a solution of KSeCN (0.288 g, 2.0 mmol) in dry acetone (10 cm^3^) at room temperature. The mixture was stirred at room temperature for 3 h, then 2,4,6-tri-tert-butylaniline (0.523 g, 2.0 mmol) was added in a pot. The suspension was stirred in the darkness at room temperature for another 18 h. To the above solution was added 2-bromo-2′,5′-dimethoxyacetophenone (0.258 g, 1.0 mmol), and the mixture was stirring at room temperature for 24 h, then refluxed for 3 h. Upon cooling to room temperature and removing the solvent, the residue was penetrating with water and extracted with dichloromethane (30 cm^3^ × 3), combined organic layers and dried over MgSO_4_. After removing solvent, the final residue was purified by silica gel column (eluted by 1:5 ethyl acetate–hexane) to give the title product **6** as pale green paste (0.540 g, 81%). Selected IR (KBr, cm^−1^): 1619, 1476, 1434, 1395, 1362, 1237, 1020, 880, 770, 642, 616. ^1^H-NMR (CD_2_Cl_2_, δ), 10.22 (s, 1H), 8.33 (d, *J*(H,H) = 8.8 Hz, 2H), 8.20 (d, *J*(H,H) = 8.8 Hz, 2H), 8.16 (s, 1H), 8.13 (s, 2H), 7.22 (d, *J*(H,H) = 8.5 Hz, 1H), 6.94 (d, *J*(H,H) = 8.5 Hz, 1H), 3.80 (s, 3H), 3.79 (s, 3H), 3.72 (s, 3H), 2.54 (s, 2H), 1.39–1.21 (m, 27H) ppm. ^13^C-NMR (CD_2_Cl_2_, δ), 167.4, 164.1, 163.1, 153.9, 153.8, 149.7, 148.5, 148.1, 132.7, 132.3, 130.2, 128.7, 123.4, 122.3, 120.8, 113.6, 56.6, 56.5, 56.0, 39.6, 37.3, 35.4, 32.8, 32.4, 31.9, 31.4 ppm. ^77^Se-NMR (CD_2_Cl_2_, δ), 590.7 ppm. HRMS (CI^+^, *m/z*): found 681.2795 [M + H]^+^, calculated mass for C_37_H_48_N_2_O_5_SeH: 681.2798. Elemental analysis: Found C, 65.24; H, 7.53; N, 4.05; C_37_H_48_N_2_O_5_Se requires C, 65.28; H, 7.26; N, 4.12. 

#### 3.2.4. Synthesis of 1,4-bis[*N*-(4-(4-methoxyphenyl)-3-(4-(pentafluoro-l^6^-sulfanyl)phenyl)-1,3-selenazol-2(3*H*)-ylidene)]terephthalamide (**7**)

To a solution of terephthaloyl dichloride (0.203 g, 1.0 mmol) in 30 cm^3^ of dry acetone was added dropwise a solution of KSeCN (0.288 g, 2.0 mmol) in dry acetone (10 cm^3^) at room temperature. The mixture was stirred at room temperature for 3 h, then 4-pentafluorosulfanylaniline (0.438 g, 2.0 mmol) was added, the suspension was stirred in the darkness at room temperature for another 18 h. To the above mixture 4-methoxybenzoyl bromide (0.458 g, 2.0 mmol) was added and the mixture was reflux for 2 h. Upon cooling to room temperature and removing the solvent, the residue was penetrated with water and extracted with dichloromethane (30 cm^3^ × 3), the combined organic layers and dried over MgSO_4_. After removing the solvent, the final residue was purified by silica gel column (eluted by 1:5 (*v*/*v*) ethyl acetate–hexane) to give the title product **7**. Yellow solid (0.450 g, 43%). M.p. 124–126 °C. Two diastereoisomers were found in ca. 3:2 intensity ratio. Selected IR (KBr, cm^−1^): 1675, 1599, 1507, 1456, 1408, 1325, 1293, 1251, 1175, 1099, 1031, 1015, 895, 833, 660, 593, 584, 578. ^1^H-NMR (CD_2_Cl_2_, δ), 8.20 (d, *J*(H,H) = 8.1 Hz, 2H), 8.09 (s, 1H), 7.96 (d, *J*(H,H) = 8.7 Hz, 2H), 7.90 (d, *J*(H,H) = 8.3 Hz, 2H), 7.86 (d, *J*(H,H) = 8.8 Hz, 2H), 7.79 (d, *J*(H,H) = 9.1 Hz, 2H), 7.59 (d, *J*(H,H) = 8.7 Hz, 2H), 7.45 (d, *J*(H,H) = 8.4 Hz, 2H), 7.25 (s, 1H), 7.09 (d, *J*(H,H) = 8.5 Hz, 2H), 6.98 (d, *J*(H,H) = 8.8 Hz, 2H), 6.82 (d, *J*(H,H) = 8.7 Hz, 2H), 6.81 (d, *J*(H,H) = 8.9 Hz, 2H), 6.65 (d, *J*(H,H) = 8.7 Hz, 2H), 3.90 (s, 3H), 3.80 (s, 3H) ppm. ^13^C-NMR (CD_2_Cl_2_, δ), 173.9, 173.4, 165.9, 165.4, 160.1, 160.0, 141.7, 141.0, 139.6, 139.4, 136.9, 136.2, 131.8, 130.6, 130.4, 129.5, 129.2, 128.9, 128.7, 127.1, 126.9, 126.5, 126.4, 124.0, 119.5, 117.5, 114.0, 113.9, 113.6, 113.3, 109.9, 109.6, 55.5, 55.3 ppm. ^19^F-NMR (CD_2_Cl_2_, *δ*), 85.1 (pentet, ^2^*J*(F,F) = 150.2 Hz, 1F, S-F_ax_), 63.2 (d, ^2^*J*(F,F) = 150.2 Hz, 4F, S-F_eq_) ppm. ^77^Se-NMR (CD_2_Cl_2_, δ), 617.7 and 614.3 ppm. HRMS (CI^+^, *m*/*z*): found 1042.9795 [M + H]^+^, calculated mass for C_40_H_28_F_10_N_4_O_4_S_2_Se_2_H: 1042.9798. Elemental analysis: Found C, 46.18; H, 2.46; N, 5.23; C_40_H_28_F_10_N_4_O_4_S_2_Se_2_ requires C, 46.16; H, 2.71; N, 5.38.

## 4. Conclusions

In conclusion, we have found that aroyl chlorides react with potassium selenocycnate, followed by treatment in situ with bulky aniline, 1,2,4-tri-tert-butylaniline, leading to the expected selenoamides and unusual diselenazoles. Further selenation of the selenoamide by using Woollins’ Reagent gives a new selenoformamide and nucleophilic addition of selenoureas with acyl bromides affords the unexpected carbamimidoselenoates rather than the expected 1,3-selenazoles. All new compounds were fully characterised by spectroscopic methods and X-ray crystal structure analyses. The strong intramolecular N–H···O hydrogen bonding and the weak intramolecular C–H···Se hydrogen bonding contributed for the supramolecular structure in selenoamide **2**. The weak intermolecular C–H∙∙∙O and C–H∙∙∙Se interactions led to the formation of supramolecular framework in **3**. Further stabilization of the crystal structures in compounds **4** and **6** is achieved by strong intramolecular N–H∙∙∙O hydrogen bonding.

## Figures and Tables

**Table 1:** Details of the X-ray data collections and refinements for compounds **2**–**6**.

Compound	2·CHCl_3_	3	4	5	6
Formula	C_27_H_36_Cl_3_N_3_O_3_Se	C_26_H_33_N_3_O_2_Se_2_	C_36_H_45_N_3_O_6_Se	C_19_H_31_NSe	C_37_H_48_N_2_O_5_Se
*M*	635.92	577.49	694.73	352.42	679.76
T/K	173	125	173	125	173
Crystal system	triclinic	triclinic	triclinic	orthorhombic	monoclinic
Space group	*P* $\bar{1}$	*P* $\bar{1}$	*P* $\bar{1}$	*Pnma*	*P*2_1_*/n*
*a/*Å	9.6580 (12)	9.4547 (7)	15.5888 (3)	20.2224 (14)	18.003 (3)
*b/*Å	10.9397 (12)	11.4917 (8)	16.3917 (4)	15.2835 (11)	9.5828 (16)
*c/*Å	15.5479 (17)	27.2316 (19)	24.7330 (10)	5.8839 (4)	21.764 (4)
α/°	91.094 (4)	93.099 (6)	71.062 (6)	90	90
β/°	91.4028 (15)	95.470 (6)	74.004 (7)	90	106.755 (4)
γ/°	113.824 (3)	90.116 (6)	62.683 (6)	90	90
*U/*A^3^	1501.5 (3)	2940.9 (4)	5250.6 (4)	1818.5 (2)	3595.3 (11)
*Z*	2	4	6	4	4
*µ/*cm^−1^	15.493	25.389	11.207	20.608	10.868
Reflections collected	27118	23358	64396	13393	42195
Independent reflections	6636	10652	19067	1718	6570
*R*_int_	0.0389	0.0979	0.0618	0.1034	0.0613
*R1* [*I* > 2σ (*I*)]	0.0294	0.0658	0.0614	0.0403	0.0429
*wR2*	0.0819	0.1741	0.1713	0.1218	0.1066

## Supplementary Materials

Supplementary materials for ^1^H and ^13^C-NMR spectra of new compounds are available online.
