# Supplementary material for: Diverse Derivatives of Selenoureas: A Synthetic and Single Crystal Structural Study
Source: Molecules. 2018 Aug 25;23(9):2143. doi: 10.3390/molecules23092143 (PMC6225220; doi:10.3390/molecules23092143)
Supplement: Supplementary file 1 [file molecules-23-02143-s001.pdf]

Supporting Information

Diverse Derivatives of Selenoureas: A Synthetic and Single Crystal Structural Study

*Guoxiong Hua, David B. Cordes, Junyi Du, Alexandra M. Z. Slawin and J. Derek Woollins**

1. ^{1}H, ^{13}C NMR spectra of compounds **2 - 7**

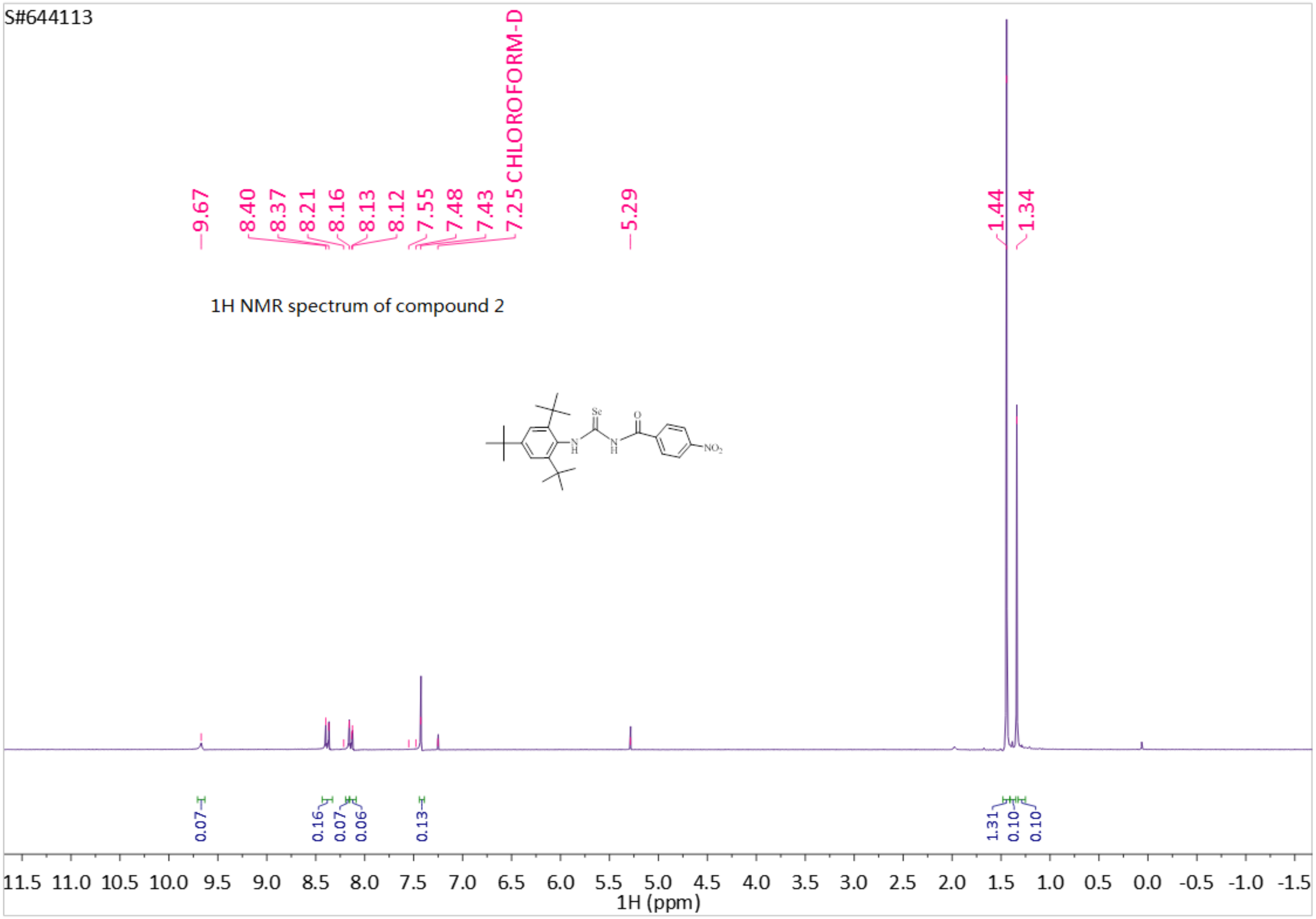

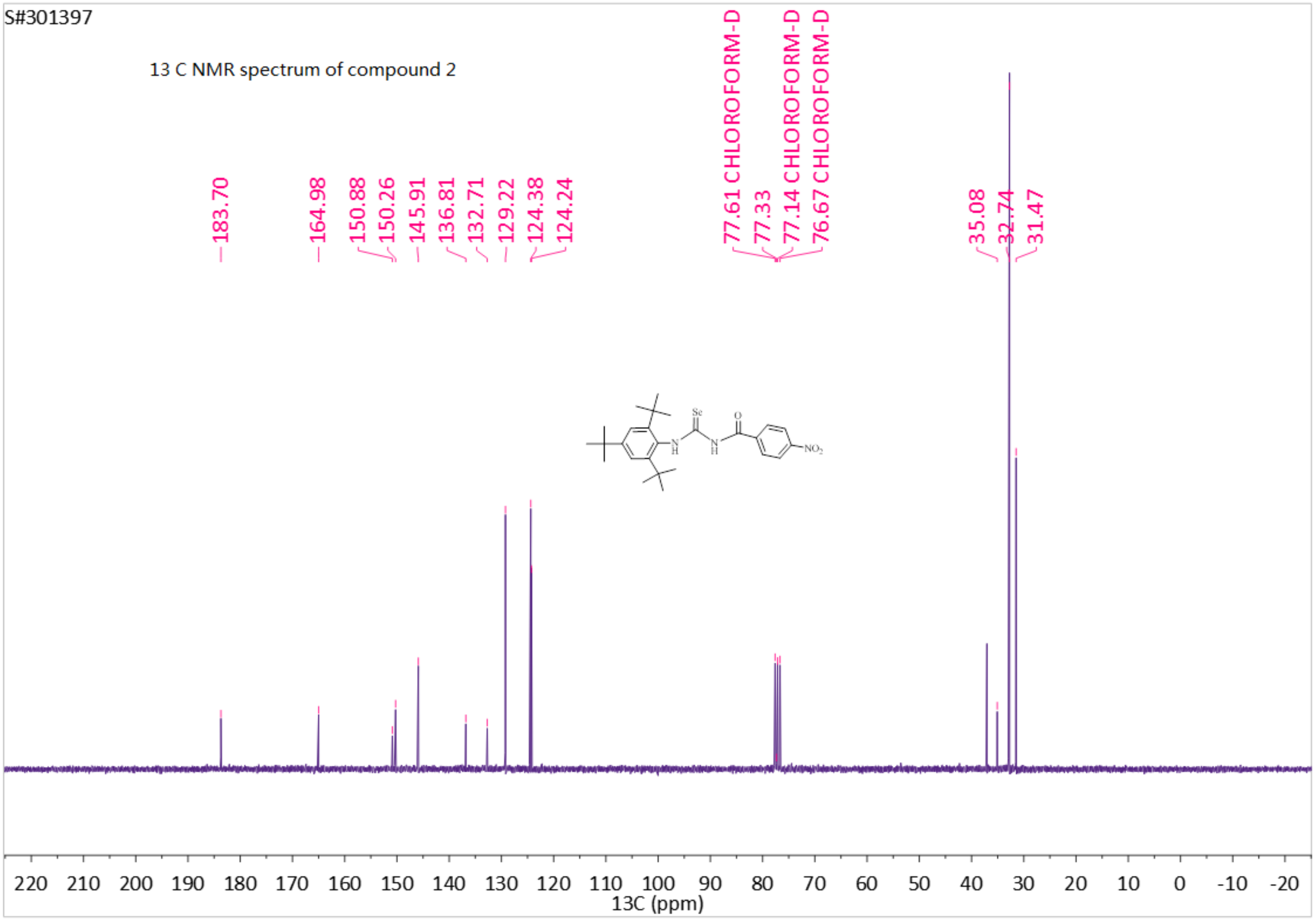
S#301397
13 C NMR spectrum of compound 2
183.70
164.98
150.88
150.26
145.91
136.81
132.71
129.22
124.38
124.24
77.61 CHLOROFORM-D
77.33
77.14 CHLOROFORM-D
76.67 CHLOROFORM-D
35.08
32.74
31.47
NO2
220 210 200 190 180 170 160 150 140 130 120 110 100 90 80 70 60 50 40 30 20 10 0 -10 -20
13C (ppm)

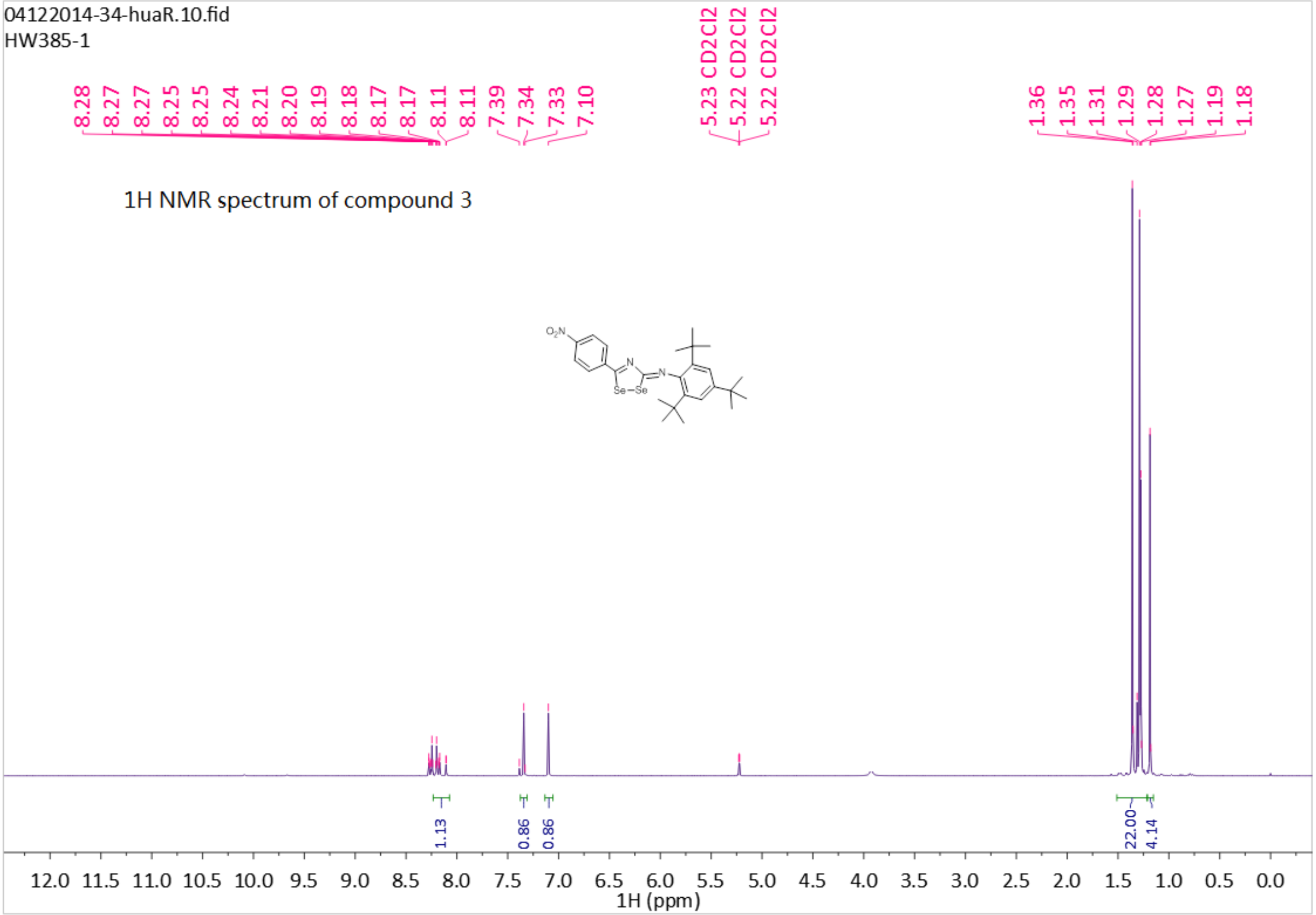
04122014-34-huaR.10.fid
HW385-1
8.28 8.27 8.27 8.25 8.25 8.24 8.21 8.20 8.19 8.18 8.17 8.17 8.11 8.11 7.39 7.34 7.33 7.10
5.23 CD2Cl2 5.22 CD2Cl2 5.22 CD2Cl2
1.36 1.35 1.31 1.29 1.28 1.27 1.19 1.18
1H NMR spectrum of compound 3
1.13 0.86 0.86 22.00 4.14
12.0 11.5 11.0 10.5 10.0 9.5 9.0 8.5 8.0 7.5 7.0 6.5 6.0 5.5 5.0 4.5 4.0 3.5 3.0 2.5 2.0 1.5 1.0 0.5 0.0
1H (ppm)

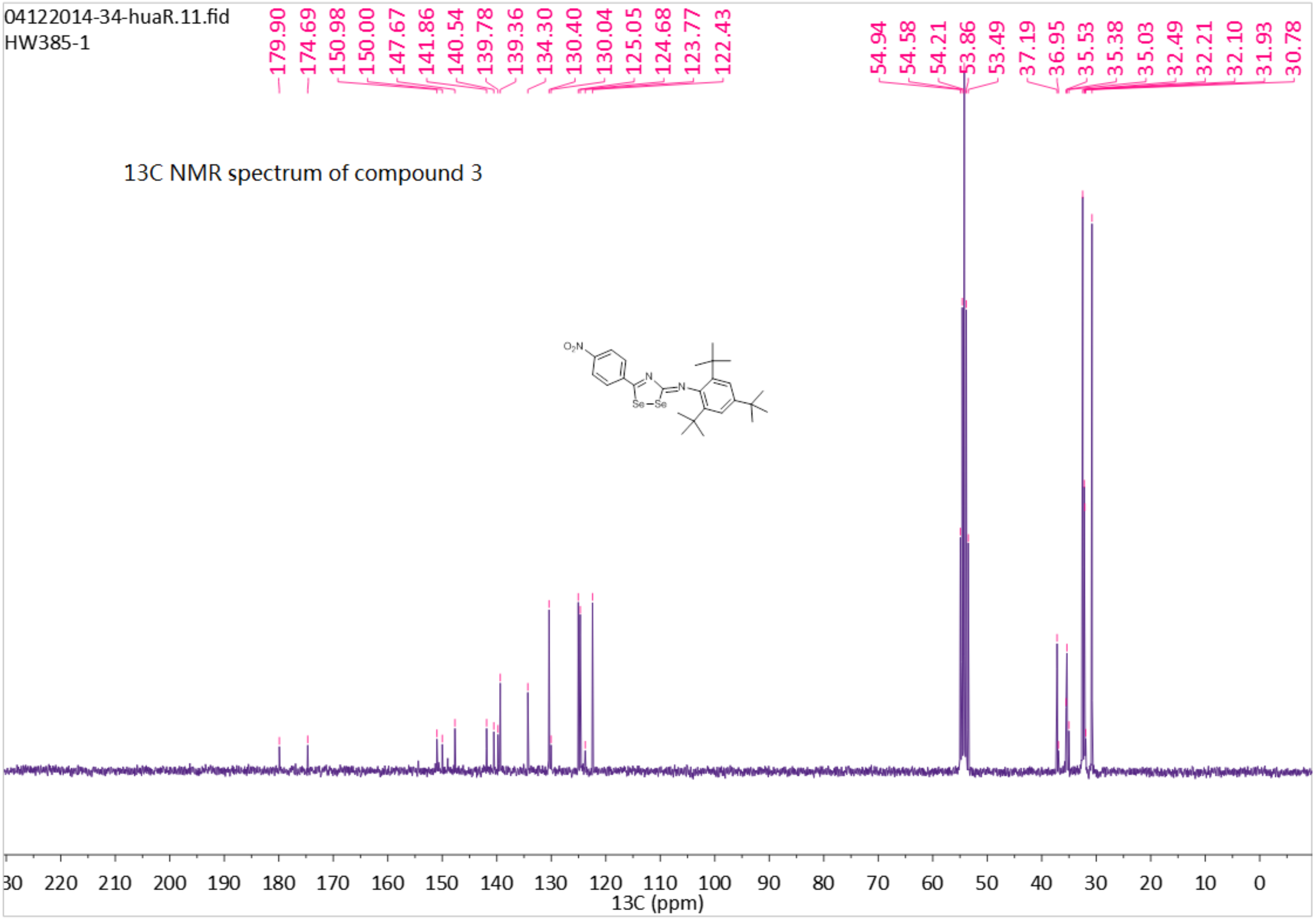

04122014-34-huaR.11.fid
HW385-1
179.90
174.69
150.98
150.00
147.67
141.86
140.54
139.78
139.36
134.30
130.40
130.04
125.05
124.68
123.77
122.43
54.94
54.58
54.21
53.86
53.49
37.19
36.95
35.53
35.38
35.03
32.49
32.21
32.10
31.93
30.78
13C NMR spectrum of compound 3
O2N
N
Se
Se
13C (ppm)

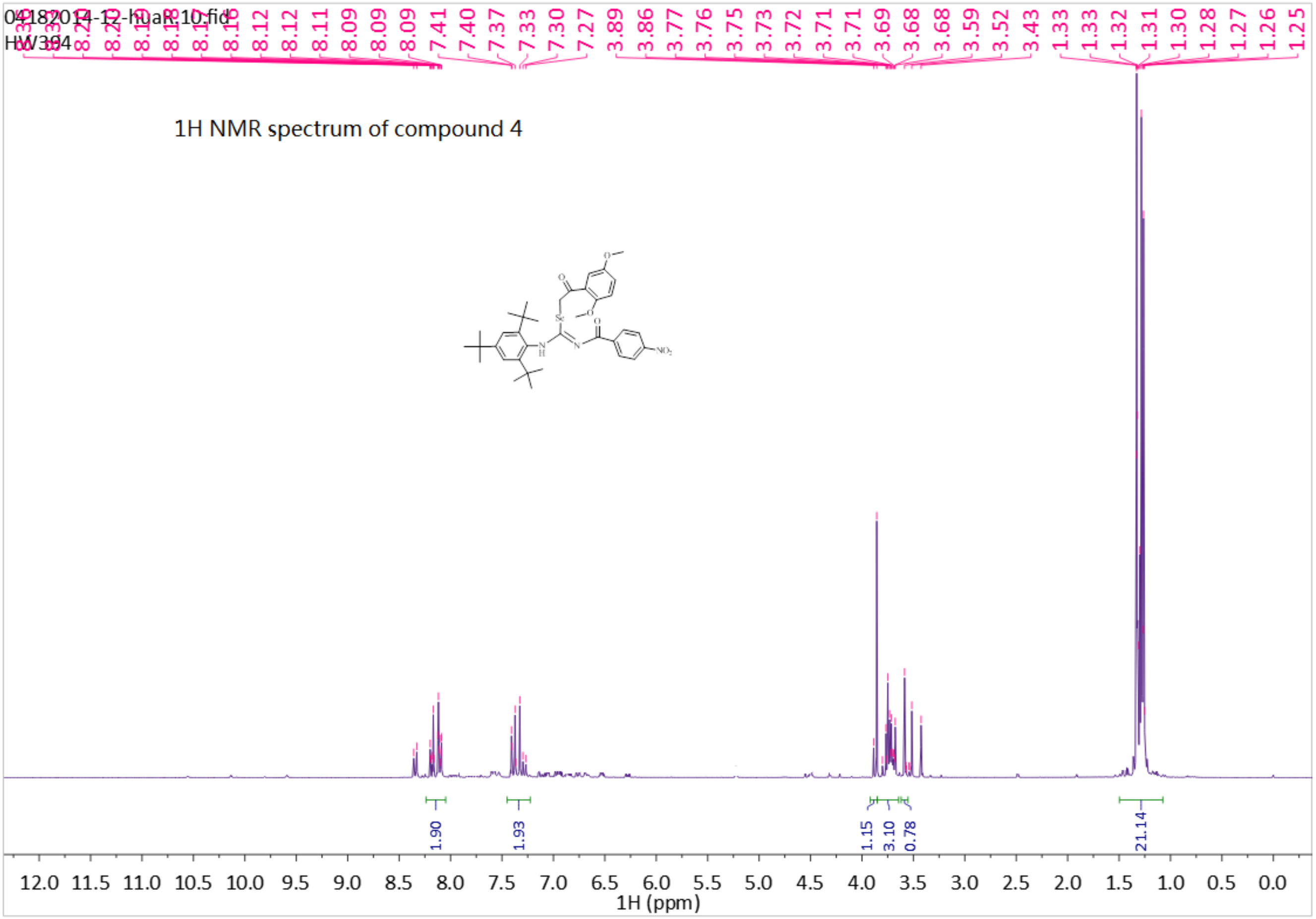
1H NMR spectrum of compound 4
HW394
1H (ppm)

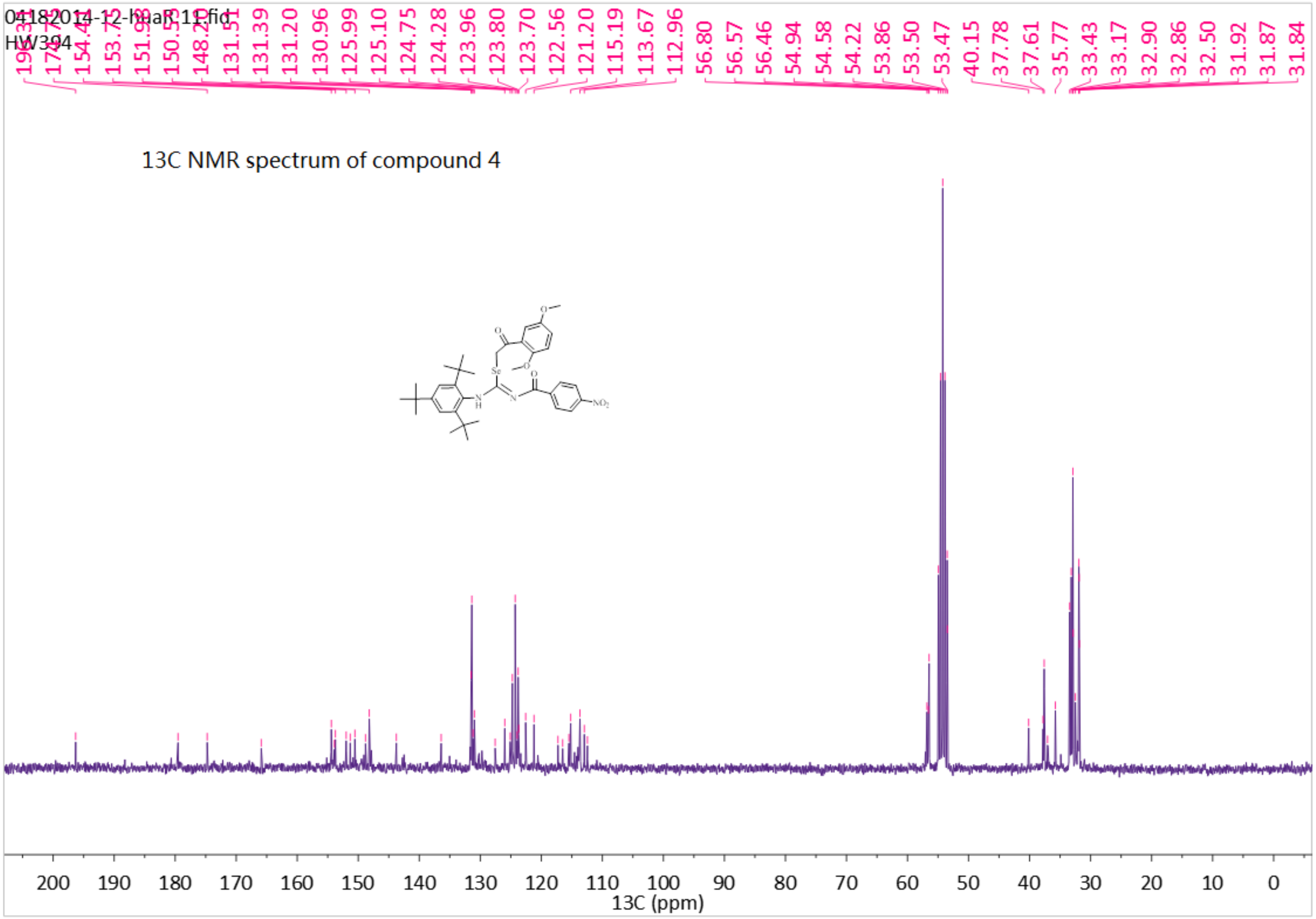
04182014-12-huan.11.fid
HW394
13C NMR spectrum of compound 4
13C (ppm)

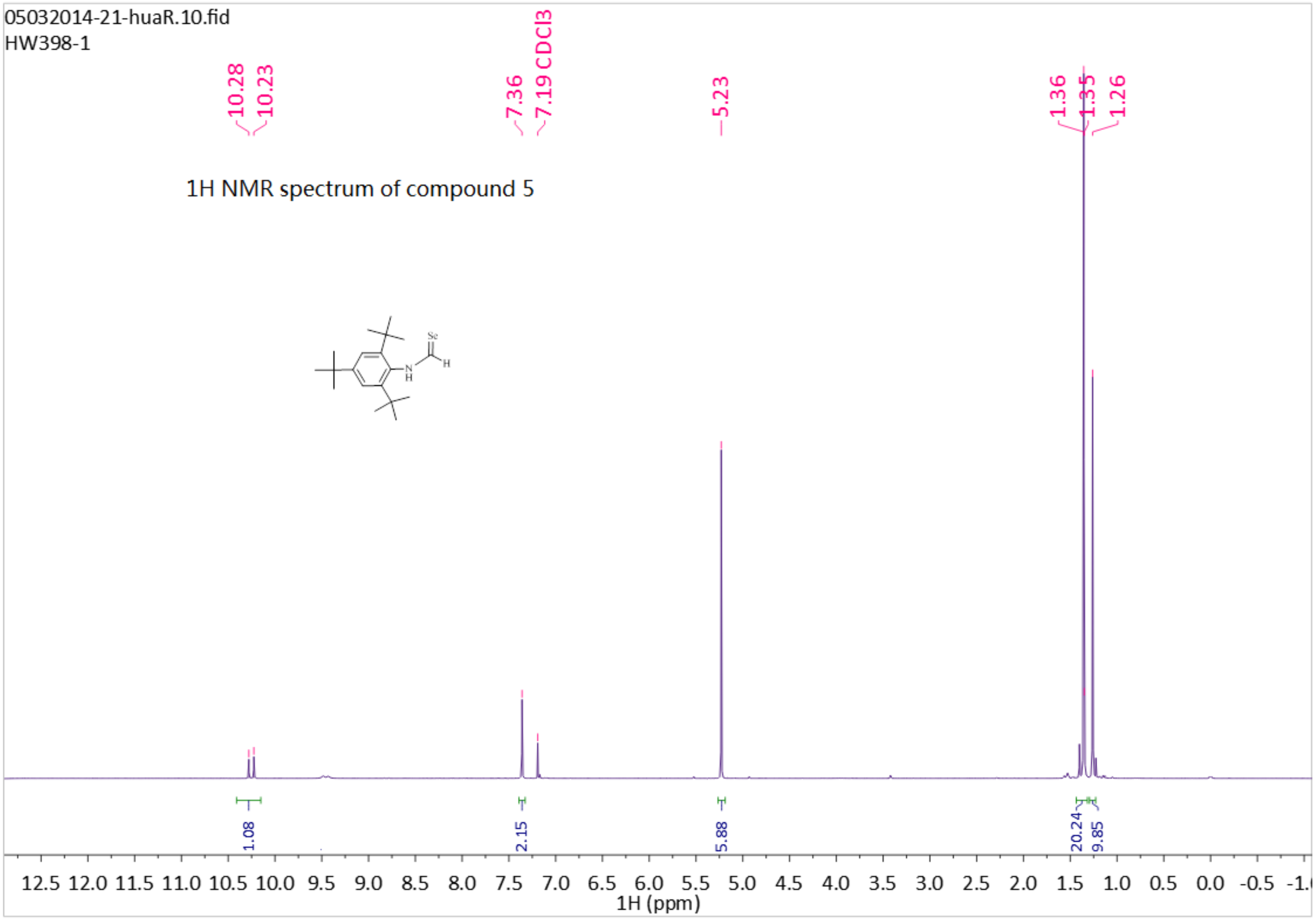

05032014-21-huaR.10.fid
HW398-1
10.28
10.23
7.36
7.19 CDCl3
5.23
1.36
1.35
1.26
1H NMR spectrum of compound 5
1.08
2.15
5.88
20.24
9.85
12.5 12.0 11.5 11.0 10.5 10.0 9.5 9.0 8.5 8.0 7.5 7.0 6.5 6.0 5.5 5.0 4.5 4.0 3.5 3.0 2.5 2.0 1.5 1.0 0.5 0.0 -0.5
1H (ppm)

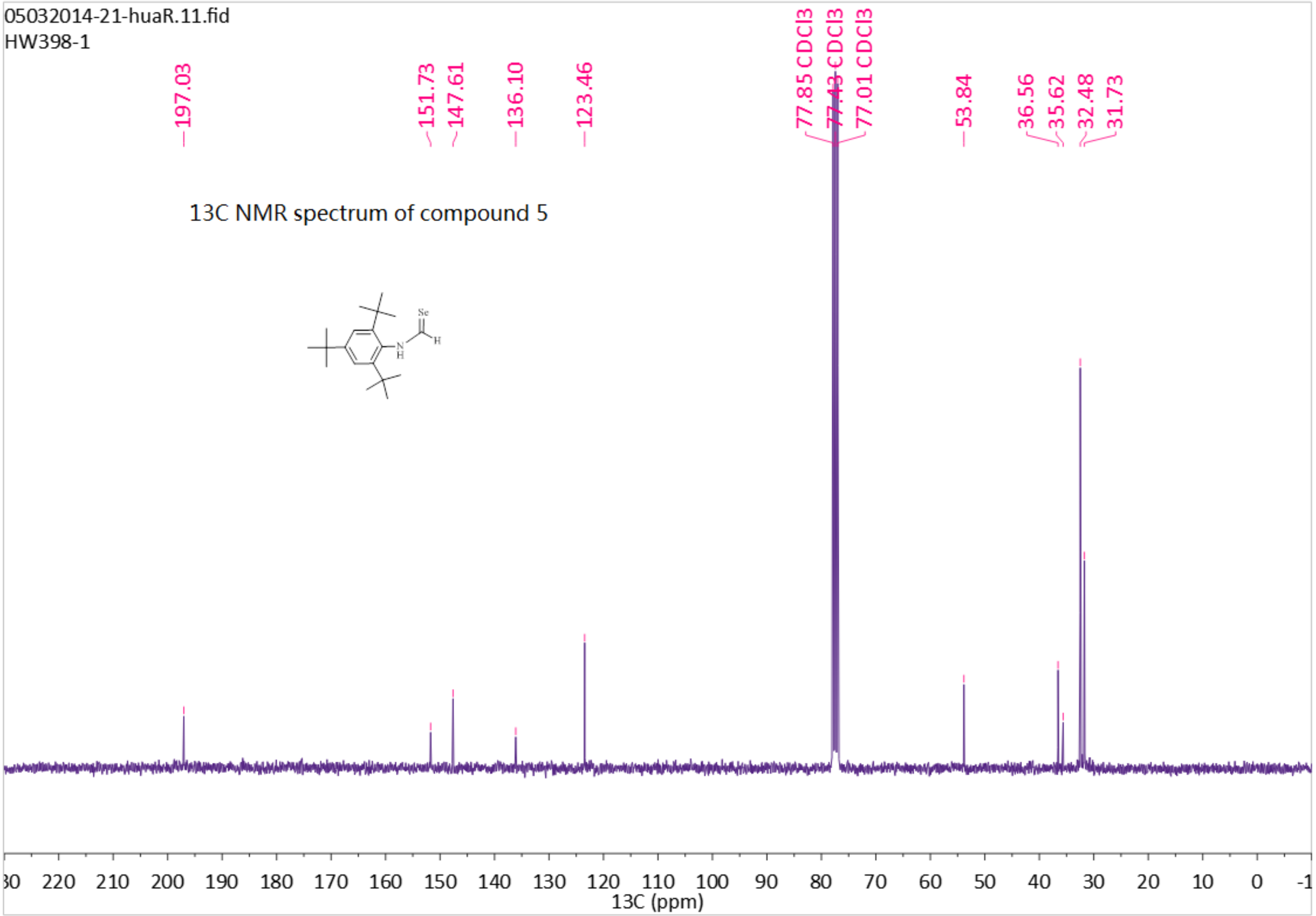
05032014-21-huaR.11.fid
HW398-1
197.03
151.73
147.61
136.10
123.46
77.85 CDCl3
77.43 CDCl3
77.01 CDCl3
53.84
36.56
35.62
32.48
31.73
13C NMR spectrum of compound 5
Se
N
H
H
220 210 200 190 180 170 160 150 140 130 120 110 100 90 80 70 60 50 40 30 20 10 0
13C (ppm)

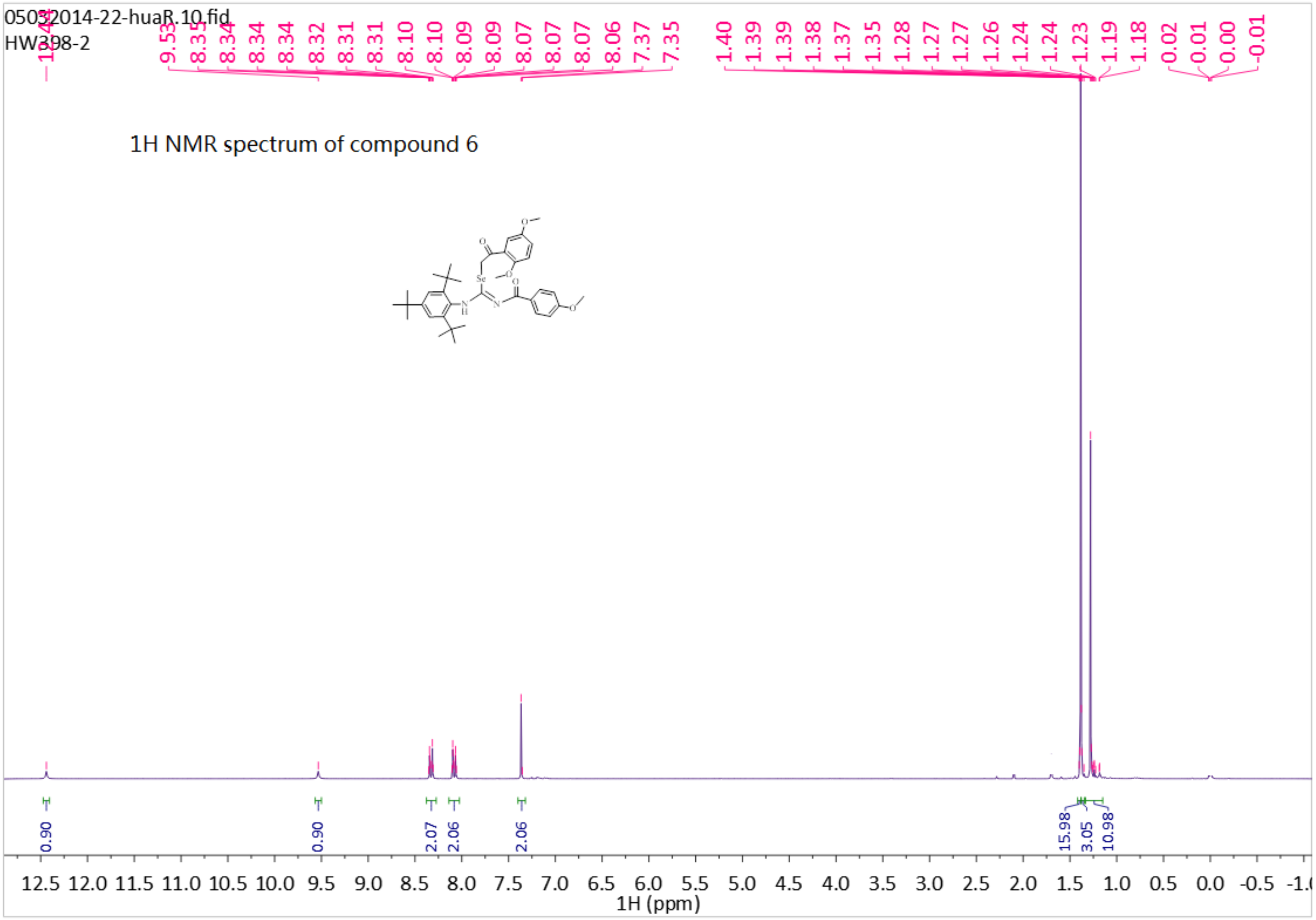
05032014-22-huaR.10.fid
HW398-2
1H NMR spectrum of compound 6
1H (ppm)

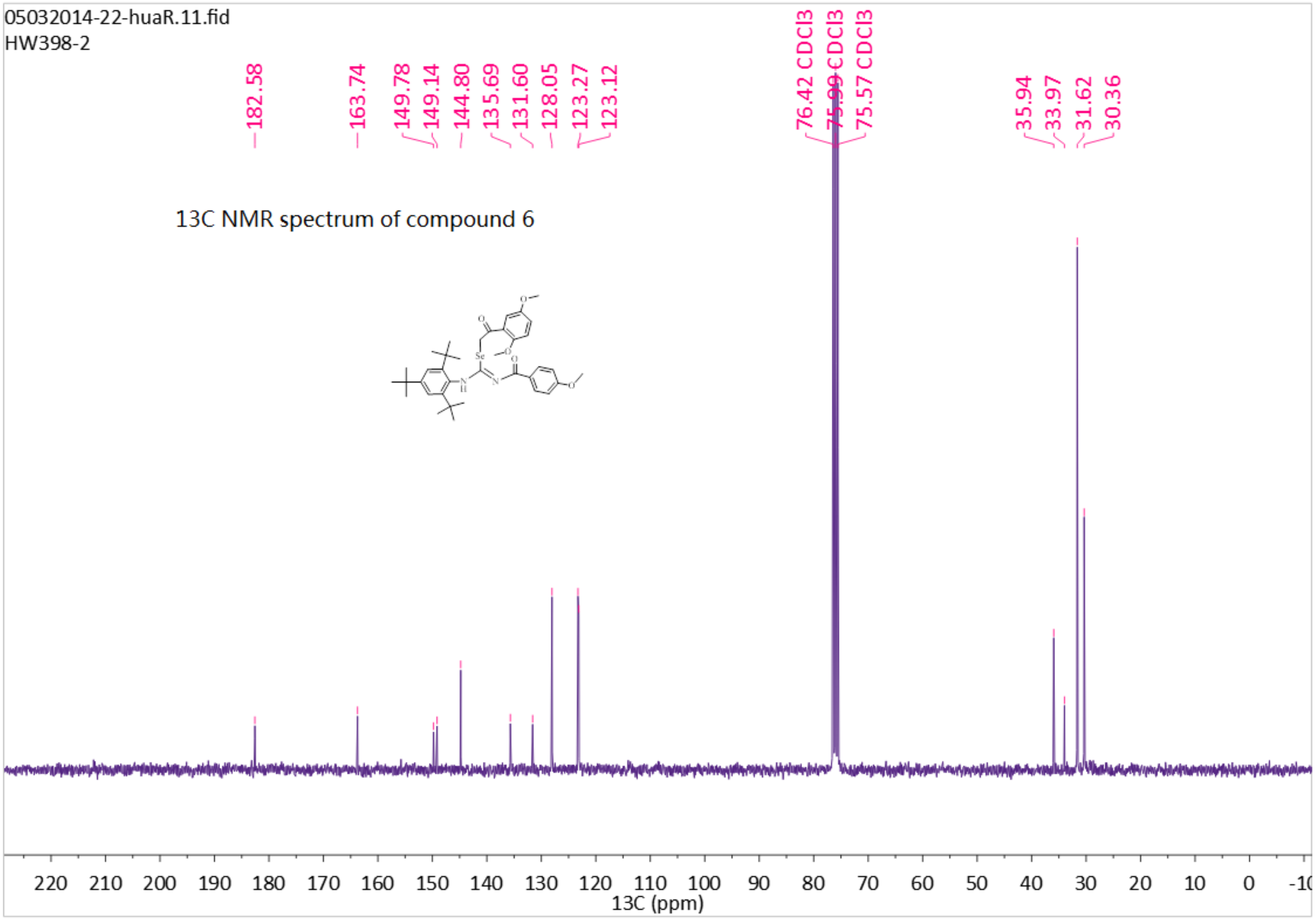
05032014-22-huaR.11.fid
HW398-2
182.58
163.74
149.78
149.14
144.80
135.69
131.60
128.05
123.27
123.12
76.42 CDCl3
75.99 CDCl3
75.57 CDCl3
35.94
33.97
31.62
30.36
13C NMR spectrum of compound 6
Se
220 210 200 190 180 170 160 150 140 130 120 110 100 90 80 70 60 50 40 30 20 10 0
13C (ppm)

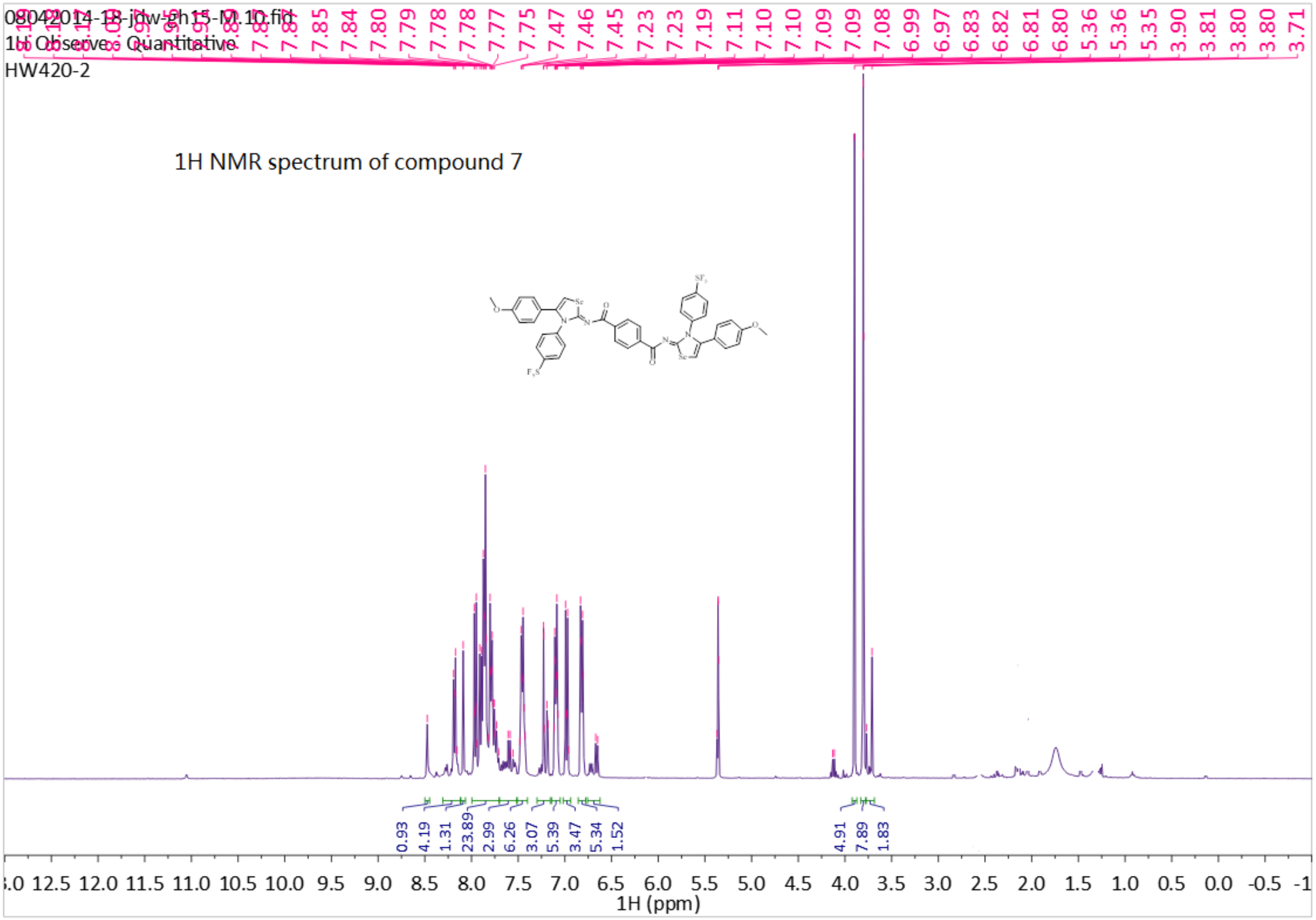
08042014-18-jdw-gh15-M.10.fid
1H Observe - Quantitative
HW420-2
1H NMR spectrum of compound 7
7.85
7.84
7.80
7.79
7.78
7.78
7.77
7.75
7.47
7.46
7.45
7.23
7.23
7.19
7.11
7.10
7.10
7.09
7.09
7.08
6.99
6.97
6.83
6.82
6.81
6.80
5.36
5.36
5.35
3.90
3.81
3.80
3.80
3.71
0.93
4.19
1.31
23.89
2.99
6.26
3.07
5.39
3.47
5.34
1.52
4.91
7.89
1.83
1H (ppm)

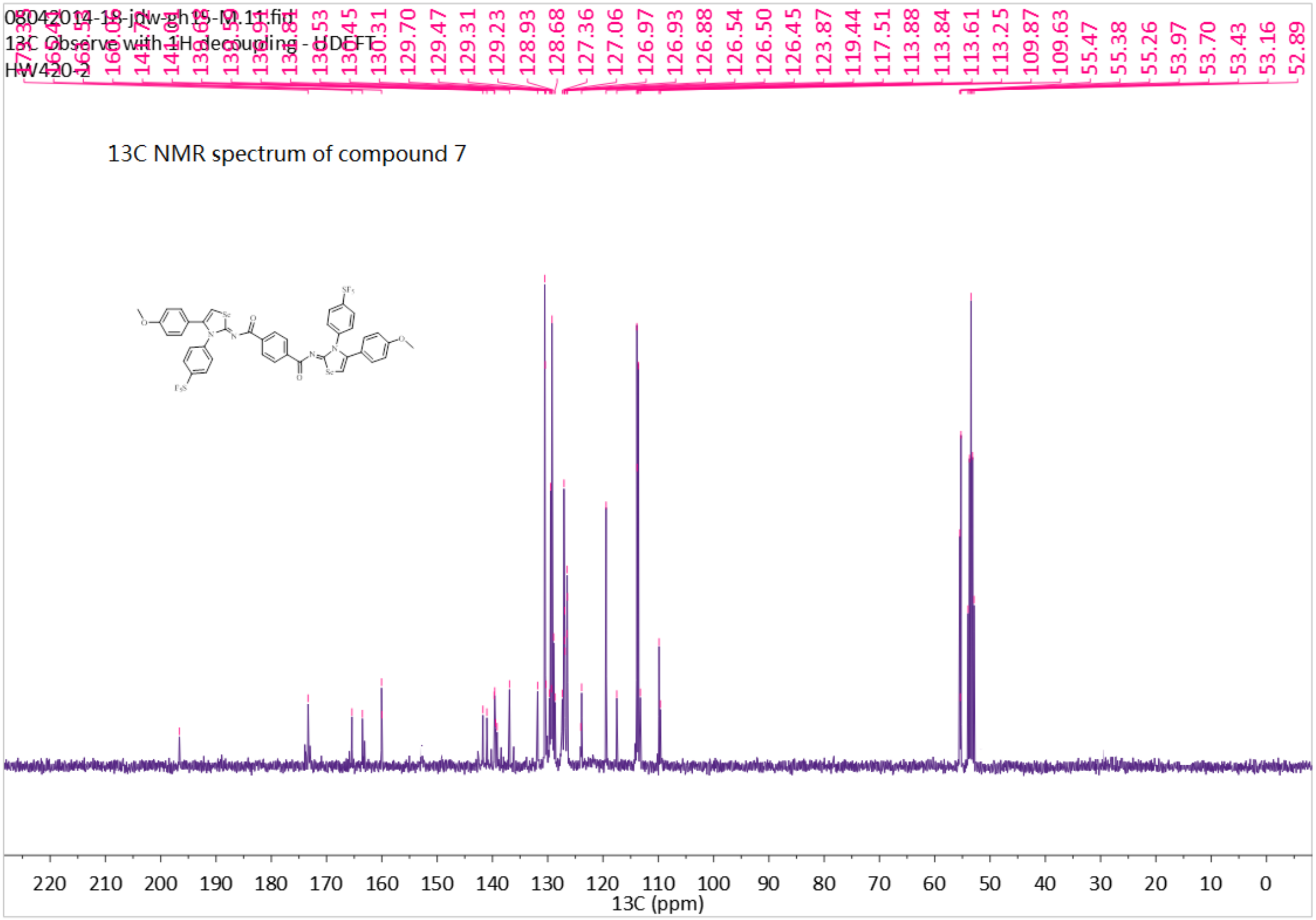
08042014-18-jdw-gh15-M.11.fid
13C Observe with 1H decoupling - UDEFT
HW420-2
13C NMR spectrum of compound 7
130.31
129.70
129.47
129.31
129.23
128.93
128.68
127.36
127.06
126.97
126.93
126.88
126.54
126.50
126.45
123.87
119.44
117.51
113.88
113.84
113.61
113.25
109.87
109.63
55.47
55.38
55.26
53.97
53.70
53.43
53.16
52.89
13C (ppm)
